# How Good Is Crude MDL for Solving the Bias-Variance Dilemma? An Empirical Investigation Based on Bayesian Networks

**DOI:** 10.1371/journal.pone.0092866

**Published:** 2014-03-26

**Authors:** Nicandro Cruz-Ramírez, Héctor Gabriel Acosta-Mesa, Efrén Mezura-Montes, Alejandro Guerra-Hernández, Guillermo de Jesús Hoyos-Rivera, Rocío Erandi Barrientos-Martínez, Karina Gutiérrez-Fragoso, Luis Alonso Nava-Fernández, Patricia González-Gaspar, Elva María Novoa-del-Toro, Vicente Josué Aguilera-Rueda, María Yaneli Ameca-Alducin

**Affiliations:** 1 Facultad de Física e Inteligencia Artificial, Universidad Veracruzana, Xalapa, Veracruz, México; 2 Centro de Investigaciones Biomédicas, Universidad Veracruzana, Xalapa, Veracruz, México; 3 Centro de Alta Tecnología de Educación a Distancia UNAM, Tlaxcala, Tlaxcala, México; Memorial Sloan Kettering Cancer Center, United States of America

## Abstract

The bias-variance dilemma is a well-known and important problem in Machine Learning. It basically relates the generalization capability (goodness of fit) of a learning method to its corresponding complexity. When we have enough data at hand, it is possible to use these data in such a way so as to minimize overfitting (the risk of selecting a complex model that generalizes poorly). Unfortunately, there are many situations where we simply do not have this required amount of data. Thus, we need to find methods capable of efficiently exploiting the available data while avoiding overfitting. Different metrics have been proposed to achieve this goal: the Minimum Description Length principle (MDL), Akaike’s Information Criterion (AIC) and Bayesian Information Criterion (BIC), among others. In this paper, we focus on crude MDL and empirically evaluate its performance in selecting models with a good balance between goodness of fit and complexity: the so-called bias-variance dilemma, decomposition or tradeoff. Although the graphical interaction between these dimensions (bias and variance) is ubiquitous in the Machine Learning literature, few works present experimental evidence to recover such interaction. In our experiments, we argue that the resulting graphs allow us to gain insights that are difficult to unveil otherwise: that crude MDL naturally selects balanced models in terms of bias-variance, which not necessarily need be the gold-standard ones. We carry out these experiments using a specific model: a Bayesian network. In spite of these motivating results, we also should not overlook three other components that may significantly affect the final model selection: the search procedure, the noise rate and the sample size.

## Introduction

It is often assumed, when collecting data of a phenomenon under investigation, that some underlying process is the responsible for the production of these data. A common approach for knowing more about this process is to build a model, from such data, that closely and reliably represents it. Once we have this model, it is potentially possible to discover the laws and principles governing the phenomenon under study and, therefore, gain a deeper understanding. Many researchers have pursued this task with very good and promising results [1]–[7]. However, a very important question arises when carrying out this task: how to choose such a model, if there are many of them, that best captures the features of the underlying process? The answer to this question has been guided by the criterion known as Occam’s razor (also called parsimony): the model that fits the data in the simplest way is the best one [1], [7]–[10]. This issue is very well known under the name of model selection [2], [3], [7], [8], [10]–[13]. The balance between goodness of fit and complexity of a model is also known as the bias-variance dilemma, decomposition or tradeoff [14]–[16].

In a nutshell, the philosophy behind model selection is to choose only one model among all possible models; this single model is treated as the “good” one and used as if it were the correct model [13]. But how can we measure the goodness of fit and complexity of the models in order to decide whether they are good or not? Different metrics have been proposed and widely accepted for this purpose: the minimum description length (MDL), the Akaike’s Information Criterion (AIC) and the Bayesian Information Criterion (BIC), among others [1]–[3], [8], [10], [13]. These metrics were designed for efficiently exploiting the data at hand while balancing bias and variance. In the context of Bayesian networks (BNs), having these measures at hand, the most intuitive and secure way to know which network is the best (in terms of this interaction) is to construct every possible structure and test each one. Some researchers [13], [17]–[20] consider the best network as the gold-standard one; i.e., the BN that generated the data. In contrast, some others [1]–[3], [5] consider that the best BN is that with the optimal balance between goodness of fit and complexity (which is not necessarily the gold-standard BN). Unfortunately, being sure that we choose the optimal-balanced BN is not, in general, feasible: Robinson [21] has shown that finding the most probable Bayesian network structure has an exponential complexity on the number of variables (Equation 1).
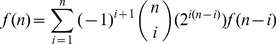
(1)


Where n is the number of nodes (variables) in the BN. If, for instance, we consider two variables, i.e., n = 2, then the number of possible structures is 3. If n = 3, the number of structures is 25; for n = 5, the number of networks is now 29, 281 and for n = 10, the number of networks is about 4.2×10^18^. In order to partially solve this complex problem, much work has been carried out on heuristic methods, namely methods that use a certain kind of reliable criterion to avoid exhaustive enumeration [9], [13], [22]–[32].

Despite this important limitation, we can evaluate the performance of these metrics in an ideal environment as well as in a realistic one. Our experiments consider each possible structure with n = 4; i.e., 543 different networks, in combination with different probability distributions and sample sizes, plotting the resulting bias-variance interaction given by crude MDL. We use the term “crude” in the sense of Grünwald’s [2]: the two-part version of MDL (Equation 3), where the term “crude” implies that code lengths for a specific model are not optimal (for more details on this, see [2]). In contrast, Equation 4 shows a refined version of MDL: it basically says that the complexity of a model does not only depend on the number of parameters but also on its functional form. Such functional form is taken into account by the third term of this equation. Since we are focusing on crude MDL, we do not give here details about refined MDL. Once again, the reader is referred to [2] for a comprehensive review. We chose to explore the crude version as this is source of contradictory results: some researchers consider that crude MDL has been specifically designed for finding the gold-standard network [13], [17]–[20], whereas others claim that, although MDL has been designed for recovering a network with a good bias-variance tradeoff (which not necessarily need be the gold-standard one), this crude version of MDL is not complete; thus, it will not work as expected [1]–[3], [5].

Our results suggest that crude MDL tends not to find the gold-standard network as the one with the minimum score but a network that optimally balances accuracy and complexity (thus recovering the ubiquitous bias-variance interaction). By accuracy we do not mean classification accuracy but the computation of the corresponding log likelihood of the data given a BN structure (see first term of Equation 3). By complexity we mean the second term of equation 3, which, in our case, is proportional to the number of arcs of the BN structure (see also Equation 3a). In terms of MDL, the lower the score a BN yields, the better. Moreover, we identify that this metric is not the only responsible for the final selection of the model but a combination of different dimensions: the noise rate, the search procedure and the sample size.

In this work, we graphically characterize the performance of crude MDL in model selection. It is important to emphasize that, although the MDL criterion and its different versions and extensions have been widely studied in the context of Bayesian networks (see Section ‘Related work’), none of these works, to the best of our knowledge, has graphically presented its corresponding empirical performance in terms of the interaction between accuracy and complexity. Thus, this is our main contribution: the illustration of the graphical performance of crude MDL for BN model selection, which allows us to more easily visualize its properties and gain more insights about it.

The remainder of the paper is organized as follows. In Section ‘Bayesian networks’, we provide a definition for Bayesian networks as well as the background of a specific problem we are focused on here: learning BN structures from data. In Section ‘The problems’, we explicitly mention the problem we are dealing with: the performance of crude MDL for model selection in the context of BN. In Section ‘Related work’, we describe some related work that studies the behavior of crude MDL in model selection. In Section ‘Material and Methods’, we present the materials and methods used in our analyses. In Section ‘Experimental methodology and results’, we explain the methodology of the experiments carried out and present the results. In Section ‘Discussion’, we discuss such results and finally, in Section ‘Conclusion and future work’, we conclude the paper and propose some directions for future work.

### Bayesian Networks

**Figure 1:**
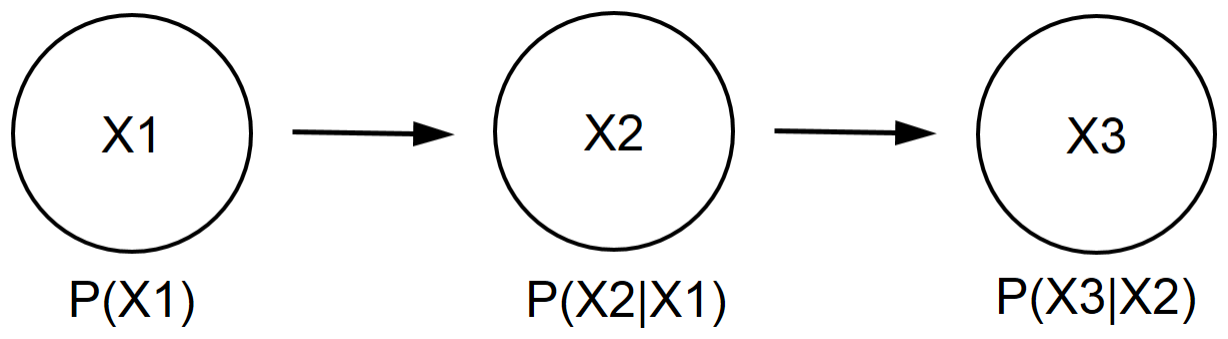
A simple Bayesian network.

A Bayesian network (BN) [9], [29] is a graphical model that represents relationships of probabilistic nature among variables of interest (Figure 1). Such networks consist of a qualitative part (structural model), which provides a visual representation of the interactions amid variables, and a quantitative part (set of local probability distributions), which permits probabilistic inference and numerically measures the impact of a variable or sets of variables on others. Both the qualitative and quantitative parts determine a unique joint probability distribution over the variables in a specific problem [9], [29], [33] (Equation 2). In other words, a Bayesian network is a directed acyclic graph consisting of [11]:

- nodes, which represent random variables; arcs, which represent probabilistic relationships among these variables and
- for each node, there exists a local probability distribution attached to it, which depends on the state of its parents.

An important concept within the framework of Bayesian networks is that of conditional independence [9], [29]. This concept refers to the case where every instantiation of a specific variable (or a set of variables) leaves other two variables independent each other. In the case of Figure 1, once we know variable X_2_, variables X_1_ and X_3_ become conditionally independent. The corresponding local probability distributions are P(X_1_), P(X_2_|X_1_) and P(X_3_|X_2_).

In sum, one of the great advantages of BNs is that they allow the representation of a joint probability distribution in a compact and economical way by making extensive use of conditional independence, as shown in Equation 2:
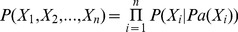
(2)where *P(X_1_, X_2_, …, X_n_)* represents the joint probability of variables *X_1_, X_2_, …, X_n_*; *Pa(X_i_)* represents the set of parent nodes of *X_i_*; i.e., nodes with arcs pointing to *X_i_* and *P(X_i_|Pa(X_i_))* represents the conditional probability of *X_i_* given its parents. Thus, Equation 2 shows how to recover a joint probability distribution from a product of local conditional probability distributions.

### Learning Bayesian Network Structures From Data

The qualitative and quantitative nature of Bayesian networks determines basically what Friedman and Goldszmidt [33] call the learning problem, which comprises a number of combinations of the following sub-problems:

- Structure learning
- Parameter learning
- Probability propagation
- Determination of missing values (also known as missing data)
- Discovery of hidden or latent variables

Since this paper focuses on the performance of MDL in the determination of the structure of a BN from data, it is only the first problem of the above list that will have further elaboration here. The reader is referred to [34] for an extensive literature review on all the above sub-problems.

Structure learning is the part of the learning problem that has to do with finding the topology of the BN; i.e., the construction of a graph that shows the dependence/independence relationships among the variables involved in the problem under study [33], [34]. Basically, there are three different ways for determining the topology of a BN: the manual or traditional approach [35], the automatic or learning approach [9], [30], in which the work presented in this paper is inspired, and the Bayesian approach, which can be seen as a combination of the previous two [13]. Friedman and Goldszmidt [33], Chickering [36], Heckerman [13], [26] and Buntine [34] give a very good and detailed account of this structure-learning problem within the automatic approach in Bayesian networks. The motivation for this approach is basically to solve the problem of the manual extraction of human experts’ knowledge found in the traditional approach. We can do this by using the data at hand collected from the phenomenon under investigation and pass them on to a learning algorithm in order for it to automatically determine the structure of a BN that closely represents such a phenomenon. Since the problem of finding the best BN is NP-complete [34], [36] (Equation 1), the use of heuristic methods is compulsory.

Generally speaking, there are two different kinds of heuristic methods for constructing the structure of a Bayesian network from data: constraint-based and search and scoring based algorithms [9], [11], [23], [29], [30], [33], [36]. We focus here on the latter. The philosophy of the search and scoring methodology has the two following typical characteristics:

- a measure (score) to evaluate how well the data fit with the proposed Bayesian network structure (goodness of fit) and
- a searching engine that seeks a structure that maximizes (minimizes) this score.

For the first step, there are a number of different scoring metrics such as the Bayesian Dirichlet scoring function (BD), the cross-validation criterion (CV), the Bayesian Information Criterion (BIC), the Minimum Description Length (MDL), the Minimum Message Length (MML) and the Akaike’s Information Criterion (AIC) [13], [22], [23], [34], [36]. For the second step, we can use well-known and classic search algorithms such as greedy-hill climbing, best-first search and simulated annealing [13], [22], [36], [37]. Such procedures act by applying different operators, which in the framework of Bayesian networks are:

- the addition of a directed arc
- the reversal of an arc
- the deletion of an arc

In each step, the search algorithm may try every allowed operator and score to create each resulting graph; it then chooses the BN structure that has more potential to succeed, i.e., the one having the highest (lowest) score. In order for the search procedures to work, we need to provide them with an initial BN. There are typically three different search-space initializations: an empty graph, a complete graph or a random graph. The search-space initialization chosen determines which operators can be firstly used and applied.

In sum, search and scoring algorithms are a widely used option for learning the structure of a Bayesian network from data; many of them have used MDL as a score metric with good results [17]–[20], [24]. However, as we shall see in the next section, we find some problems that at first sight seem to do with the definition of the MDL metric itself. Also, we find different works that are inconsistent each other with respect to their findings regarding the performance of MDL as a metric for model selection. In the following sections, we present these inconsistencies.

### The Problems

**Figure 2:**
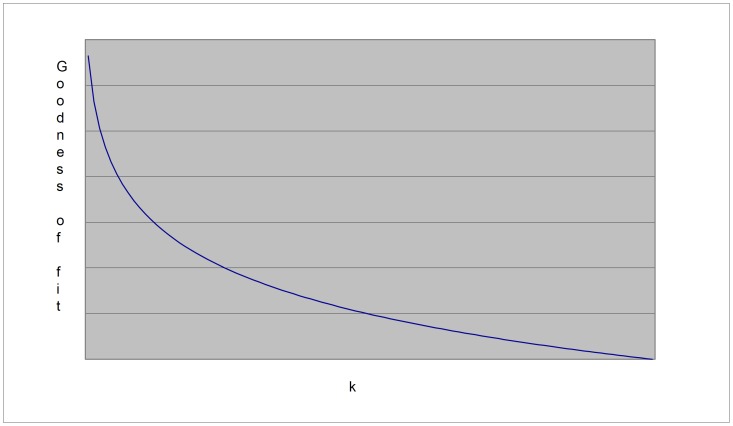
The first term of MDL.

**Figure 3:**
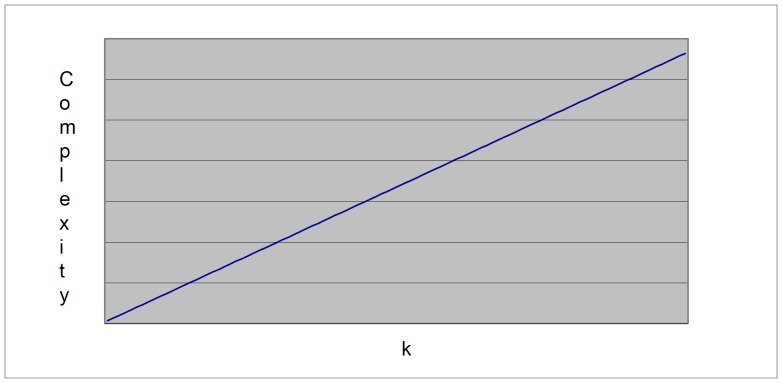
The second term of MDL.

**Figure 4:**
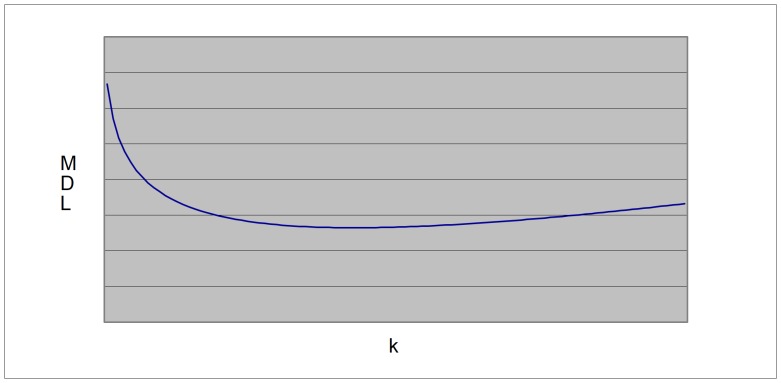
The MDL graph.

Let us first consider the traditional or crude definition of MDL (Equation 3) [2], [13]:
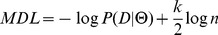
(3)where *D* is the data, 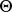 represents the parameters of the model, *k* is the dimension of the model (number of free parameters) and *n* is the sample size. The parameters 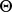 of our specific model are the corresponding local probability distributions for each node in the network. Such distributions are determined by the structure of the BN (for a clear example, see [34]). The way to compute *k* (the dimension of the model) is given in Equation 3a.
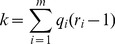
(3a)where m is the number of variables, qi is the number of possible configurations of the parents of variable Xi and ri is the number of values of that variable. For details on how to compute Equation 3 in the context of BN, the reader is referred to [34]. The first term of this equation measures the accuracy (log likelihood) of the model (Figure 2); i.e., how well it fits the data, whereas the second term measures the complexity (Figure 3): such a term punishes models more heavily as they get more complex. In our case, the complexity of a BN is, in general, proportional to the number of arcs (given by k in Equation 3a) [17]. In theory, metrics that incorporate these two terms can identify models with a good balance between accuracy and complexity (Figure 4).

Regarding the first term of MDL (Figure 2), Grünwald [2], [3] notes an important analogy between codes and probability distributions: a large probability means a small code and vice versa. To be clearer about this, a probability of 1 will produce a code of length 0 and a probability approaching 0 will produce a code of length approaching ∞. In order to build the graph in Figure 2, we just compute the first term of Equation 3 by giving probability values in the range (0–1].

In this figure, the X-axis represents *k* (Equation 3a), which, in general, is proportional to the number of arcs in a BN. The Y-axis is –log P(*D*|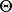) (the accuracy term), which is the log likelihood of the data given the parameters of the model. Since the log likelihood is used as the accuracy term, such a term is better as it approaches zero. As can be seen, while a BN becomes more complex (in terms of *k*), its accuracy gets better (i.e., the log likelihood approaches zero). Unfortunately, such a situation is not desirable since the resulting model will, in general, overfit unseen data. This behavior is similar to that when only the training set is used for both the construction of a model and the test of this model [16]. By definition, MDL has been explicitly designed for finding models with a good tradeoff between accuracy and complexity [1]–[3], [5]. Unfortunately, the first term alone does not achieve this goal. That is why we need a second term: a term that punishes the complexity of a model (Figure 3). In order to build the graph in this figure, we just compute the second term of Equation 3 by giving complexity values in the arbitrary range [0–1].

The X-axis represents *k* too, while the Y-axis represents the complexity. Hence, the second term punishes complex models more heavily than it does to simpler models. This term is used for compensating the training error. If we only take into account such a term, we do not get well-balanced BNs either since this term alone will always choose the simplest one (in our case, the empty BN structure – the network with no arcs). Therefore, MDL puts these two terms together in order to find models with a good balance between accuracy and complexity (Figure 4) [17]. In order to build the graph in this figure, we now compute the interaction between accuracy and complexity, where we manually assign small values of *k* to large code lengths and vice versa, as MDL dictates.

It is important to notice that this graph is also the ubiquitous bias-variance decomposition [16]. On the X-axis, *k* is again plotted. On the Y-axis, the MDL score is now plotted. In the case of MDL values, the lower, the better. As the model gets more complex, the MDL gets better up to a certain point. If we continue increasing the complexity of the model beyond this point, the MDL score, instead of improving, gets worse. It is precisely in this lowest point where we can find the best-balanced model in terms of accuracy and complexity (bias-variance). However, this ideal procedure does not easily tell us how difficult would be, in general, to reconstruct such a graph with a specific model in mind. To appreciate this situation in our context, we need to see again Equation 1. In other words, an exhaustive analysis of all possible BN is, in general, not feasible. But we can carry out such an analysis with a limited number of nodes (say, up to 4 or 5) so that we can assess the performance of MDL in model selection. One of our contributions is to clearly describe the procedure to achieve the reconstruction of the bias-variance tradeoff within this limited setting. To the best of our knowledge, no other paper shows this procedure in the context of BN. In doing so, we can observe the graphical performance of MDL, which allows us to gain insights about this metric. Although we have to bear in mind that the experiments are carried out using such a limited setting, we will see that these experiments are enough to show the mentioned performance and generalize to situations where we may have more than 5 nodes.

As we will see with more detail in the next section, there is a discrepancy on the MDL formulation itself. Some authors claim that the crude version of MDL is able to recover the gold-standard BN as the one with the minimum MDL, while others claim that this version is incomplete and does not work as expected. For instance, Grünwald and other researchers [1], [5] claim that model selection procedures incorporating Equation 3 will tend to choose complex models instead of simpler ones. Thus, from these contradictory results, we have two more contributions: a) our results suggest that crude MDL produces well-balanced models (in terms of bias-variance) and that these models do not necessarily coincide with the gold-standard BN, and b) as a corollary, these findings imply that there is nothing wrong with the crude version.

Authors who consider that crude definition of MDL is incomplete, propose a refined version (Equation 4) [2], [3], [5]:
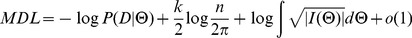
(4)where |*I*(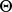)| is the determinant of the Fisher information matrix *I*(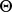) and the constant term o(1) approaches 0 as n (the sample size) approaches ∞. Roughly speaking, this formula says that the complexity of a model does not only depend on the number of parameters but also on its functional form. Such functional form is taken into account by the third term of Equation 4. Since we are considering the crude version of MDL for our experiments (Equation 3), we do not give here details of this term. The interested reader might like to see [2]. We leave as a future work the comparison between the crude version and the refined one.

### Related Work

Recall, from Sections ‘Introduction’ and ‘The problems’, that some researchers consider crude MDL as a metric specifically designed for finding the gold-standard BN structure [13], [17]–[20], whereas others claim that, although MDL has been designed for recovering a network (not necessarily the gold-standard network) with a good bias-variance tradeoff, this crude version of MDL is not complete; thus, it will not work as expected [1]–[3], [5]. Most of the most representative works dealing with the construction of BN structures from data fall in the first situation (the recovery of the gold-standard BN structure) [17]–[20], [34]. There are much fewer works dealing with the second situation: the study of crude MDL as a good metric for selecting BN structures from data with a good bias-variance tradeoff [24], [38], [39], [40]–[70]. In fact, these works explicitly mention the accuracy dimension but hardly the complexity dimension: they only show the accuracy performance of classifiers but seldom show the BN structures of such classifiers. In this paper, we concentrate on plotting the resulting structures and comparing the one chosen by MDL against the gold-standard network. It is important to mention that some works [24], [38], [39] have pointed out that networks with a good MDL are not necessarily good classifiers. For instance, Friedman et al. [24] trace the reason of this problem to the definition of MDL itself: it globally measures the error of the learned BN rather than the local error in the prediction of the class. They identify this problem in their experiments when MDL-based BN classifiers perform worse than Naïve Bayes on some databases. It is left then as future work, the evaluation of classification accuracy of the minimum models yielded by the different metrics considered here.

In this section, we try by no means to enumerate all of the works in both situations; instead, we mention the most representative ones.

### Learning BN Structures from Data

One of the first algorithms in recovering the structure of a BN from data is the well-known K2 procedure [23], which has been a source of motivation for carrying out research in this direction. There, the authors propose a metric (named CH in [26] because of their authors - Cooper and Herskovits) for building Bayesian networks given data. The main goal of the experiment they carry out is to test how well such a metric recovers the ALARM network [23]. The CH metric is then considered as a suitable measure for finding gold-standard networks. For some researchers, such as Heckerman [26], the CH metric is different to MDL since the former does not satisfy the property of likelihood equivalence (which says that the data should not help discriminate Bayesian network structures that represent the same conditional independence relationships). On the other hand, for some others, such as Suzuki [20], CH is similar to MDL (see below). Thus, for those who consider CH equivalent to MDL, the former would also have to be tested as suitable for either task (finding the gold-standard network or a network with a good bias-variance balance). To the best of our knowledge, CH was specifically designed for recovering gold-standard BNs and none has evaluated its performance in selecting balanced BNs. We do not assess CH in this way either but we leave it as a future work.

The work by Suzuki [19], [20] is also a good reference. Suzuki is one of the firsts in introducing the MDL metric for learning Bayesian networks from data. In both papers, he derives the MDL formula, which is similar to that in Equation 3. In fact, the only difference is that Suzuki does take into account O(1) terms. According to Grünwald [2], such terms have to be necessarily considered since they can be quite important in practice for an accurate model selection. He also points out that this equation holds only in the case when the dimension of the model (*k*) is kept fixed and the sample size tends to infinity. Thus, in that sense, it is incomplete. Even Suzuki’s MDL formulation (which takes into account O(1) terms) is incomplete for it does not consider the functional form of the model (see Equation 4). One of the most salient results in [20] is the conclusion that the CH metric (used by K2) is similar to the MDL metric in the sense that they only differ each other in the value assigned to their priors rather than in their approaches (Bayesian and MDL respectively). Another important result is that he concludes that the metric used by Lam and Bacchus [18] (see below) is not actually a description length, as they claim, for it does not satisfy Kraft’s inequality [19]. It is worth noting that Suzuki points out there that the term log *n* (in Equation 3) can be replaced with any function c(*n*) of *n*, where MDL refers to the case where c(*n*) = log *n* and AIC refers to the case where c(*n*) = 2. With this last choice, AIC is no longer MDL-based but it might perform better than MDL: an assertion that Grünwald would not agree with. However, Suzuki does not present experiments that support this claim. On the other hand, the experiments he carries out are to support that MDL can be useful in the recovery of gold-standard networks since he uses the ALARM network for this purpose: this represents a contradiction according again to Grünwald and Myung [1], [5] for, they claim, MDL has not been specifically designed for finding the true model. Furthermore, in his 1999 paper [20], Suzuki does not either present experiments in order to support his theoretical results regarding the behavior of MDL. In our experiments we empirically show that MDL does not, in general, recover gold-standard networks but networks with a good compromise between bias and variance.

Bouckaert [17] extends the K2 algorithm in the sense of using a different metric: the MDL score. He calls this modified algorithm K3. His experiments have also to do with the capability of MDL for recovering gold-standard networks. Again, as in the case of the works mentioned above, K3 procedure focuses its attention on the pursuit of finding the true distribution. An important contribution of this work is that he graphically shows how the MDL metric behaves. To the best of our knowledge, this is the only paper that explicitly shows this behavior in the context of BN. However, this graphical behavior is only theoretical rather than empirical.

The work by Lam and Bacchus [18] deals with learning Bayesian belief nets based on, they claim, the MDL principle (see criticism by Suzuki [20]). There, they conduct a series of experiments to demonstrate the feasibility of their approach. In the first set of experiments, they show that their MDL implementation is able to recover gold-standard nets. Once again, such results contradict those by Grünwald’s and ours, which we present in this paper. In the second set of experiments, they use the well-known ALARM belief network structure and compare the learned network (using their method) against it. The results show that this learned net is close to the ALARM network: there are only two extra arcs and three missing arcs. This experiment also contradicts Grünwald’s MDL concept since their goal here is to show that MDL is able to recover gold-standard networks. In the third and final set of experiments, they use only one network varying the conditional probability parameters. Then, they carry out an exhaustive search and obtain the best MDL structure given by their procedure. In one of these cases, the gold-standard network was recovered. It seems here that one important ingredient for the MDL procedure to work properly is the amount of noise in the data. We investigate such an ingredient in our experiments. In our opinion, Lam and Bacchus’s best contribution is the search algorithm that seeks for networks that minimize cross-entropy: such algorithm is not a standard hill-climbing procedure. Our results (see Sections ‘Experimental methodology and results’ and ‘Discussion’) suggest that one possibility of the MDL’s limitation in learning simpler Bayesian networks is the nature of the search algorithm.

Other important work to consider in this context is that by Van Allen et al. [unpublished data]. According to these authors, there are many algorithms for learning BN structures from data, which are designed to find the network that is closer to the underlying distribution. This is typically measured in terms of the Kullback-Leibler (KL) distance. In other words, all these procedures seek the gold-standard model. There they report an interesting set of experiments. In the first one, they carry out an exhaustive search for *n* = 5 (*n* being the number of nodes) and measure the Kullback-Leibler (KL) divergence between 30 gold-standard networks (from which samples of size 8, 16, 32, 64 and 128 are generated) and different Bayesian network structures: the one with the best MDL score, the complete, the independent, the maximum error, the minimum error and the Chow-Liu networks. Their findings suggest that MDL is a successful metric, around different midrange complexity values, for successfully handling overfitting. These findings also suggest that in some complexity values, the minimum MDL networks are equivalent (in the sense of representing the same probability distributions) to the gold-standard ones: this finding is in contradiction to ours (see Sections ‘Experimental methodology and results’ and ‘Discussion’). One possible criticism of their experiment has to do with the sample size: it could be more illustrative if the sample size of each dataset were larger. Unfortunately, the authors do not provide an explanation for that selection of sizes. In the second set of experiments, the authors carry out a stochastic study for *n* = 10. Because of the practical impossibility to perform an exhaustive search (see Equation 1), they only consider 100 different candidate BN structures (including the independent and complete networks) against 30 true distributions. Their results also confirm the expected MDL’s bias for preferring simpler structures to more complex ones. These results suggest an important relationship between sample size and the complexity of the underlying distribution. Because of their findings, the authors consider the possibility to more heavily weigh the accuracy (error) term so that MDL becomes more accurate, which in turn means that larger networks can be produced. Although MDL’s parsimonious behavior is the desired one [2], [3], Van Allen et al. somehow consider that the MDL metric needs further complication.

In another work by Van Allen and Greiner [6], they carry out an empirical comparison of three model selection criteria: MDL, AIC and Cross-Validation. They consider MDL and BIC as equivalent each other. According to their results, as the sample size grows, the MDL criterion tends to find the true network as the model with the minimum MDL: this contradicts our findings in the sense of not finding the true network (see Sections ‘Experimental methodology and results’ and ‘Discussion’). Furthermore, when they test MDL with lower entropy distributions (local probability distributions with values 0.9 or 0.1), their experiments show that MDL has a high bias for simplicity, in accordance with investigations by Grünwald and Myung [1]–[3], [5]. As can be inferred from this work, Van Allen and Greiner think MDL is not behaving as expected, for it should find the ideal structure, in contrast to what Grünwald et al. consider as a suitable behavior of such a metric. Our results support those by the latter: MDL prefers simpler networks than the true models even when the sample size grows. Also, the results by Van Allen and Greiner indicate that AIC behaves different from MDL, in contrast to our results: AIC and MDL find the same minimum network; i.e., they behave equivalently to each other.

In a seminal paper by Heckerman [13], he points out that BIC = −MDL, implying that these two measures are equivalent each other: this clearly contradicts the results by Grünwald et al. [2]. Furthermore, in two other works by Heckerman et al. and Chickering [26], [36], they propose a metric called BDe (Bayesian Dirichlet likelihood equivalent), which, in contrast to the CH metric, considers that data cannot help discriminate Bayesian networks where the same conditional independence assertions hold (likelihood equivalence). This is also the case of MDL: structures with the same set of conditional independence relations receive the same MDL score. These researchers carry out experiments to show that the BDe metric is able to recover gold-standard networks. From these results, and the likelihood-equivalence between BDe and MDL, we can infer that MDL is also able to recover these gold-standard nets. Once again, this result is in contradiction to Grünwald’s [1]–[3] and ours. On the other hand, Heckerman et al. mention two important points: 1) not only is the metric relevant for getting good results but also the search method and 2) the sample size has a significant effect on the results.

Regarding the limitation of traditional MDL for classification purposes, Friedman and Goldszmidt come up with an alternative MDL definition that is known as local structures [71]. They redefine this traditional MDL metric incorporating and exploiting the notion of a feature called CSI (context-specific independence). In principle, such local models perform better as classifiers than their global counterparts. However, this last approach tends to produce more complex networks (in terms of the number of arcs), which, according to Grünwald, do not reflect the very nature of MDL: the production of models that well balance accuracy and complexity.

It is also important to mention the work by Kearns et al. [4]. They present a beautiful theoretical and experimental comparison of three model selection methods: Vapnik’s Guaranteed Risk Minimization, Minimum Description Length and Cross-Validation. They carry out such a comparison using a particular model, called the intervals model selection problem, which is a rare case where training error minimization is possible. In contrast, procedures such as backpropagation neural networks [37], [72], whose heuristics have unknown properties, cannot achieve training error minimization. Their most significant findings have to do with the impossibility of always reducing the generalization error by diminishing the training error: this implies that there is no universal relation between these two types of error leading to either the undercoding or overcoding of data by penalty-based procedures, such as MDL, BIC or AIC. Their experimental results give us a clue for considering more than just the metric for obtaining balanced models: a) the sample size and b) the amount of noise in the data.

To close this section, it is important to recall the distinction that Grünwald and some other researchers emphasize regarding crude and refined MDL [1], [5]. For these researchers crude MDL is not complete; hence, it cannot produce well-balanced models. This assertion also applies to metrics such as AIC and BIC since they do not either take into account the functional form of the model (see Equation 4). On the other hand, there are some works, which regard BIC and MDL as equivalent [16], [40], [73]–[84]. In this paper, we also assess the performance of AIC and BIC to recover the bias-variance tradeoff. Our results suggest that, under certain circumstances, these metrics behave similarly to crude MDL.

### Learning BN Classifiers from Data

Some investigations have used MDL-like metrics for building BN classifiers from data [24], [38], [39], [40]–[70]. They partially characterize the bias-variance dilemma: their results have mainly to do with the classification performance but little to do with the structure of those classifiers. Here, we mention some of those well-known works.

A classic and pioneer work is that by Chow and Liu [41]. There, they approximate discrete probability distributions using dependence trees, which are applied to recognize (classify) hand-printed numerals. Although the method for building such trees does not strictly use an MDL-like metric but mutual information, the latter can be identified as an important part of the former. These dependence trees can be considered as a special case of a BN.

Friedman and Goldszmidt [42] present an algorithm, based on MDL, which discretize continuous attributes while learning BN classifiers. In fact, they only show accuracy results but do not show the structure of such classifiers.

Another reference work is that by Friedman et al. [24]. There, they compare the classification performance among different classifiers: Naïve Bayes, TAN (tree augmented Naïve Bayes), C4.5 and unrestricted Bayesian networks. This last type of classifiers is built using as a scoring function the MDL metric (using the same definition as in Equation 3). Although Bayesian networks are more powerful than the Naïve Bayes classifier, in the sense of more richly representing the dependences among attributes, the former perform worse on some datasets from the UCI repository [http://www.ics.uci.edu/~mlearn/MLRepository.html] than the latter, in terms of classification accuracy. Friedman et al. trace the reason of this problem to the definition of MDL itself: it globally measures the error of the learned BN rather than the local error in the prediction of the class. In other words, a Bayesian network with a good MDL score does not necessarily represent a good classifier. Unfortunately, the experiments they present in their paper are not specifically designed to prove whether MDL is good at finding the gold-standard networks. However, we can infer so from the text: “…with probability equal to one the learned distribution converges to the underlying distribution as the number of samples grows” [24]. This contradicts our experimental findings. In other words, our findings show that MDL does not in general recover the true distribution (represented by the gold-standard net) even when the sample size grows.

Cheng and Greiner [43] compare different BN classifiers: Naïve Bayes, Tree Augmented Naïve Bayes (TAN), BN Augmented Naïve Bayes (BAN) and General BN (GBN). TAN, BAN and GBN all use conditional independence tests (based on mutual information and conditional mutual information) to build their respective structure. It can be inferred from this work that such structures, combined with data, are used for classification purposes. However, these structures are not explicitly shown in this paper making it virtually impossible to measure their corresponding complexity (in terms of the number of arcs). Once again, as in the case of Chow and Liu’s work [41], these tests are not exactly MDL-based but can be identified as an important part of this metric.

Grossman and Domingos [38] propose a method for learning BN classifiers based on the maximization of conditional likelihood instead of the optimization of the data likelihood. Although the results are encouraging, the resulting structures are not presented either. If those structures were presented, that would give us the opportunity of grasping the interaction between bias and variance. Unfortunately, this is not the case.

Drugan and Wiering [75] introduce a modified version of MDL, called MDL-FS (Minimum Description Length for Feature Selection) for learning BN classifiers from data. However, we cannot measure the bias-variance tradeoff since the results these authors present are only in terms of classification accuracy. This same situation happens in Acid et al. [40] and Kelner and Lerner [39].

## Materials and Methods

### Datasets

For the tests carried out in this work, we generated databases from random 4-node gold-standard Bayesian networks with various sample sizes. All the random variables considered in these experiments are binary: this choice does not produce any significant qualitative impact on the results; rather, it makes the computation and analyses easier [6]. The use of simulated datasets is a common practice to evaluate the performance of heuristic algorithms that recover the structure of a BN from data [34], [36], [85]. Also, synthetic data from gold-standard BN give us the flexibility of plotting learning curves over different combinations of probability distributions and sample sizes (see cf. [4]). The only difference in our experiments is that we are carrying out an exhaustive search among all possible network structures (for n = 4) and using these simulated datasets to assess the potential of different metrics (including MDL) for recovering models that well balance accuracy and complexity. The methods used for generating the datasets from a specific BN structure, a specific probability distribution and a determined sample size are presented in the next section.

### Algorithm for Generating Directed Acyclic Graphs

**Figure 5:**
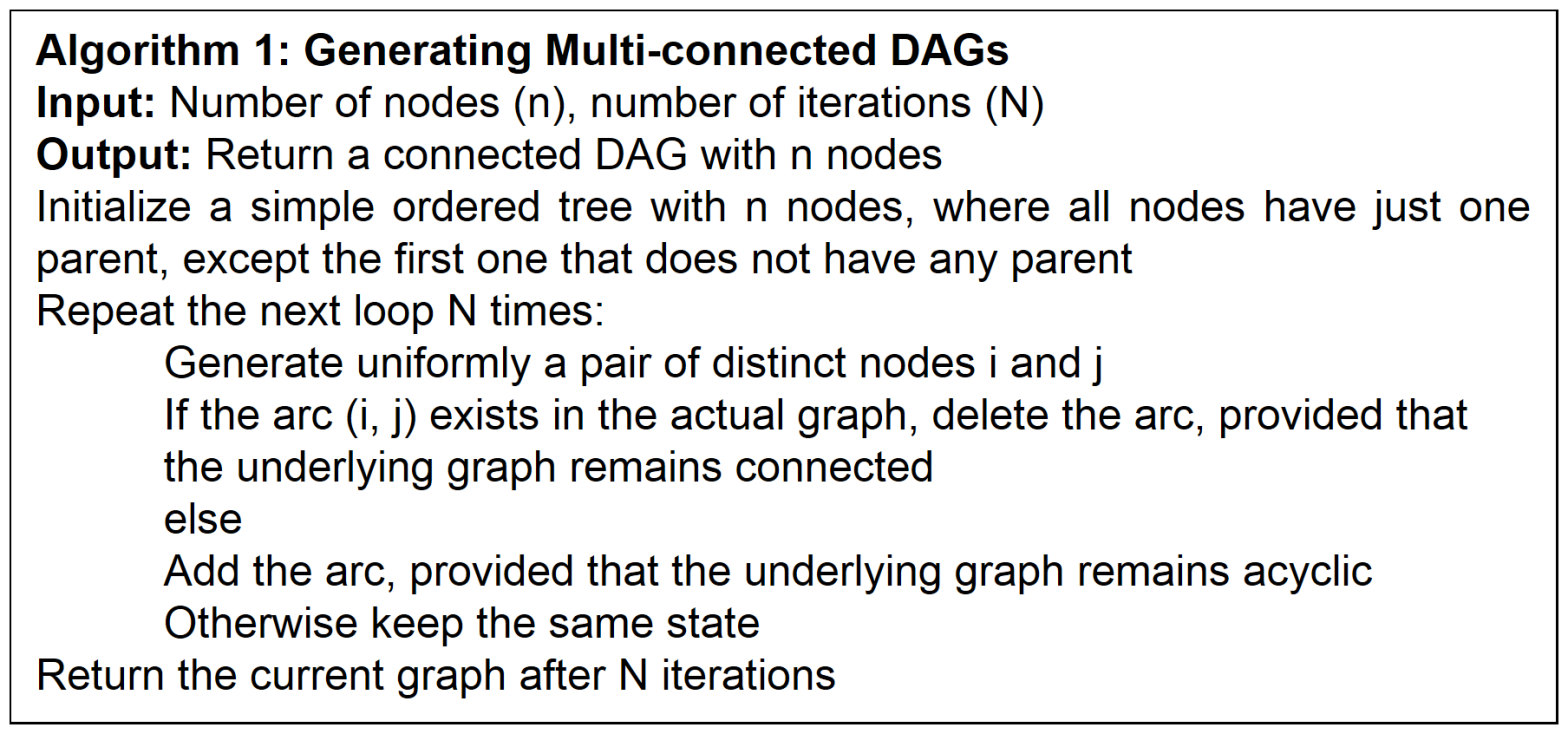
Ide and Cozman’s algorithm for generating multi-connected DAGs.

In order to generate a database, we firstly need to propose a specific structure from which such a database is created (in combination with a specific joint probability distribution and a sample size). We decided to use the procedure by Ide and Cozman [86], which allows to generate uniformly distributed DAGs. The pseudo-code of such a procedure, called algorithm 1, is given in figure 5. Note that line 01 of algorithm 1 initializes a simple ordered tree, which we achieve by using a pseudo-random number generator called ran3 [87]. It is important to mention that, although some generators can satisfy most of applications, they are not recommended as reliable random number procedures. This is because they do not either fulfill some statistical tests for randomness or cannot be used in long sequences. Since the generator we use in our experiments is based more on a subtractive method than a linear congruential one, it offers specific desirable features that the others do not: portability, low correlation in successive runs and independence on the computer arithmetic. This same procedure is used for carrying out step 03 of algorithm 1 as well. The interested reader might like to see the C code of procedure ran3 in [87].

### Generation of Conditional Probability Distributions

**Figure 6:**
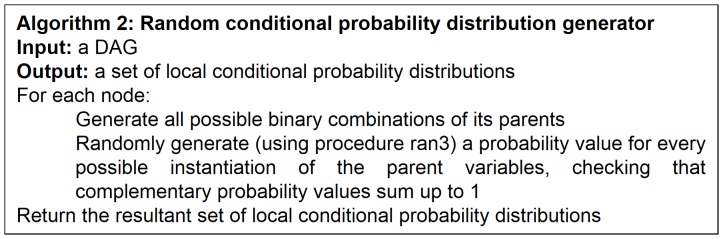
Algorithm for randomly generating conditional probability distributions.

Once we have a DAG, we randomly generate the corresponding conditional probability distributions from such a DAG using procedure ran3 as well. The pseudo-code of this random conditional probability distribution generator, which we call algorithm 2, is given in figure 6.

### Generation of Raw Sample Data

**Figure 7:**
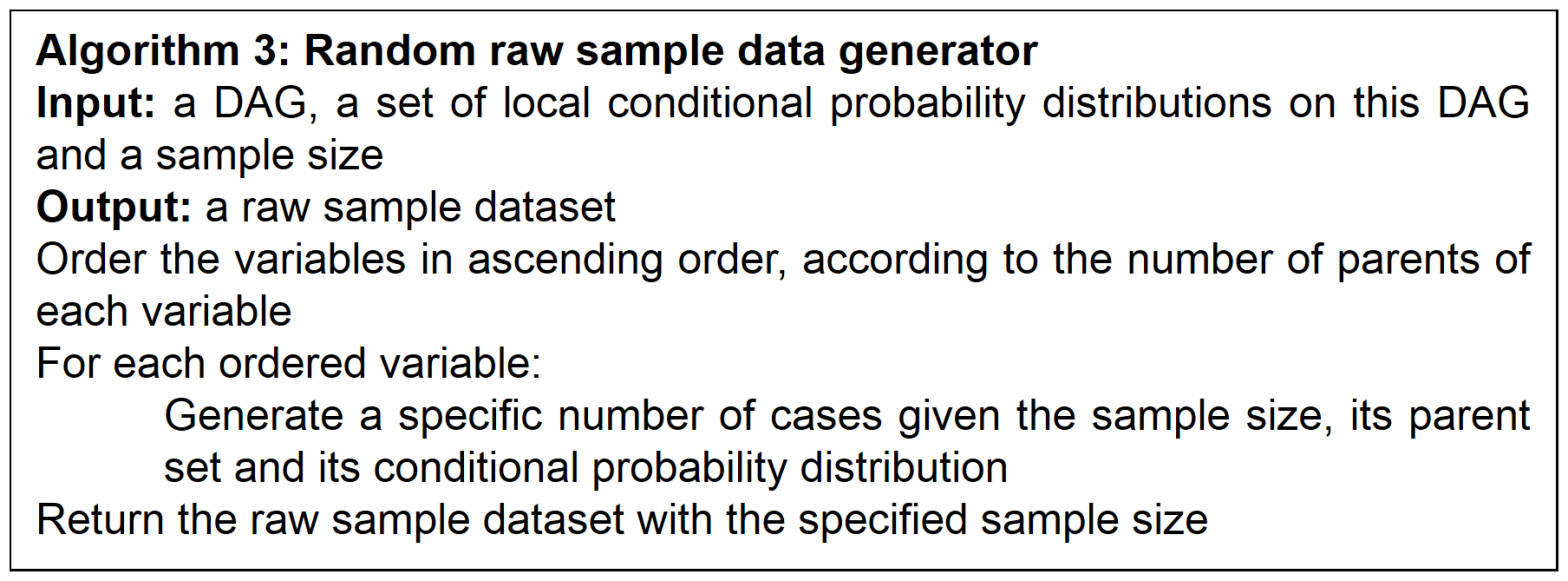
Algorithm for randomly generating raw sample data.

Given a DAG and its corresponding set of local conditional probability distributions, we generate a random data sample according to algorithm 3 (see figure 7).

### Construction of BAYESIAN Networks

**Figure 8:**
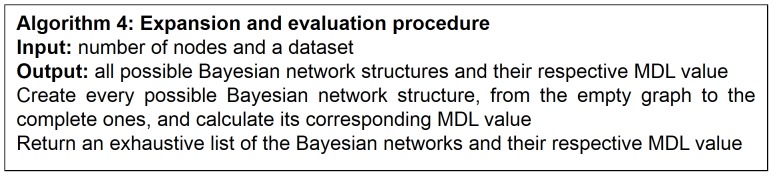
Expansion and evaluation algorithm.

Since the goal of the present study is to assess the performance of MDL (among some other metrics) in model selection; i.e., to check whether these metrics can recover the gold-standard Bayesian networks or whether they can come up with a balanced model (in terms of accuracy and complexity) that is not necessarily the gold-standard one, we need to exhaustively build all the possible network structures given a number of nodes. Recall that one of our goals is to characterize the behavior of AIC and BIC, since some works [13], [73], [88] consider them equivalent to crude MDL while others regard them different [1]–[3], [5]. For the analyses presented here, the number of nodes is 4, which produces 543 different Bayesian network structures (see equation 1). Our procedure that exhaustively builds all possible networks, called algorithm 4, is given in figure 8.

Regarding the implementation of the metrics tested here, we wrote procedures for crude MDL (Equation 3) and one of its variants (Equation 7) as well as procedures for AIC (Equations 5 and 6) and BIC (Equation 8). We included in our experiments alternative formulations of AIC and MDL (called here AIC2 and MDL2) suggested by Van Allen and Greiner [6] (Equations 6 and 7 respectively), in order to assess their performance. The justification Van Allen and Greiner provide for these alternative formulations of MDL and AIC is, for the former, that they normalize everything by 1/n (where n is the sample size) so as to compare such criterion across different sample sizes; and for the latter, they simply carry out a conversion from nats to bits by using log e.
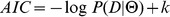
(5)




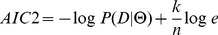
(6)




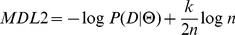
(7)




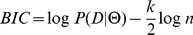
(8)


For all these equations, *D* is the data, 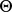 represents the parameters of the model, *k* is the dimension of the model (number of free parameters), *n* is the sample size, *e* is the base of the natural logarithm and *log e* is simply a conversion from nats to bits [6].

## Experimental Methodology and Results

In this section, we describe the experimental methodology and show the results of two different experiments. In Section ‘Discussion’, we discuss those results.

### Experiment 1

**Figure 9:**
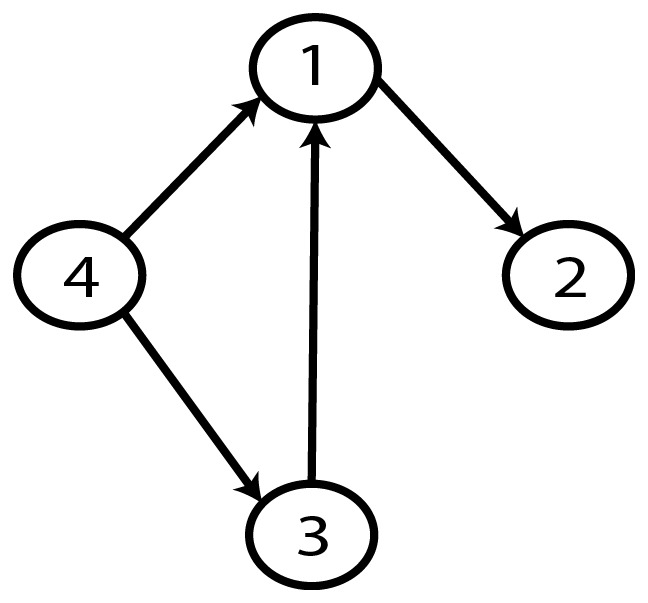
Gold-standard Network.

**Figure 10:**
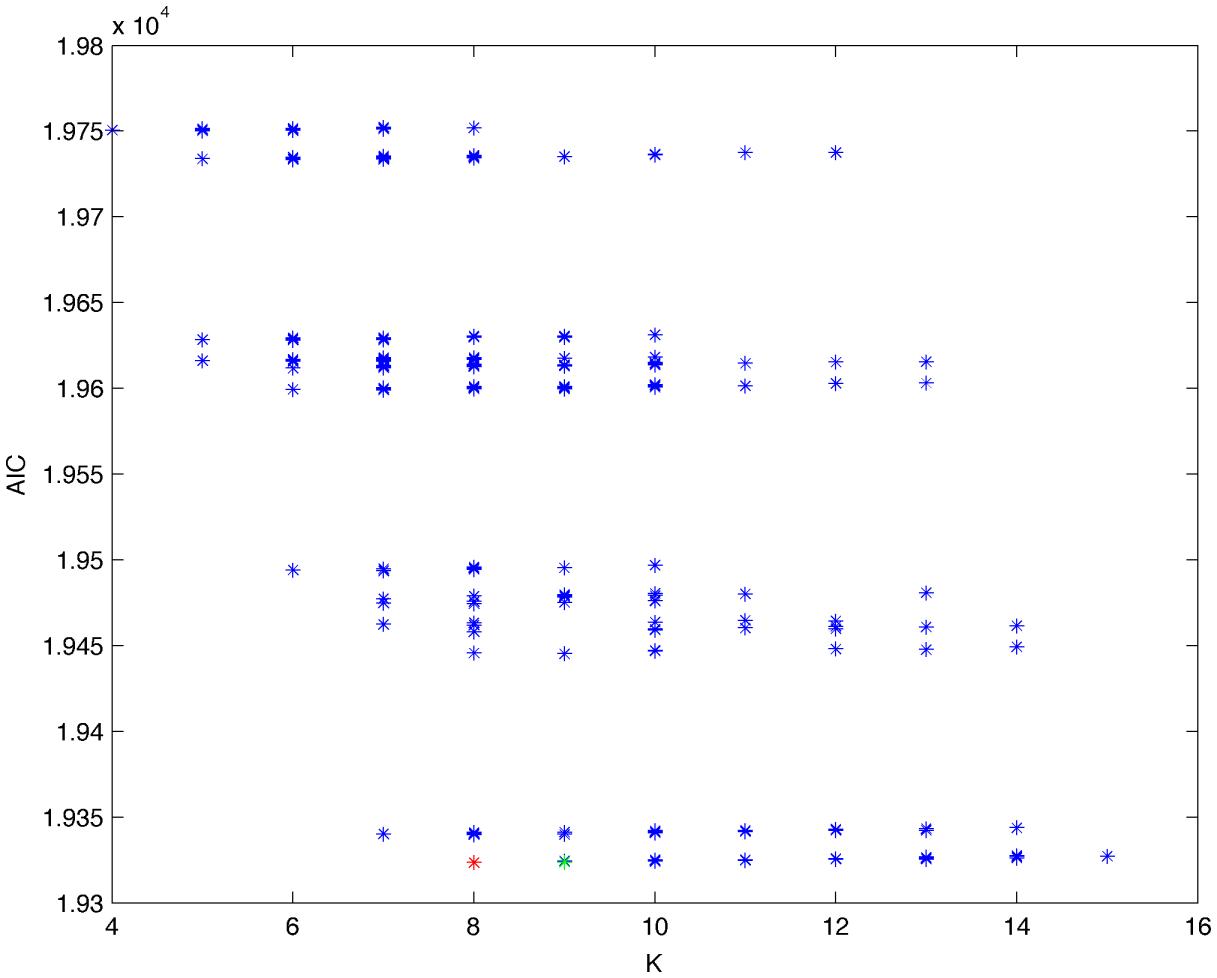
Exhaustive evaluation of AIC (random distribution).

**Figure 11:**
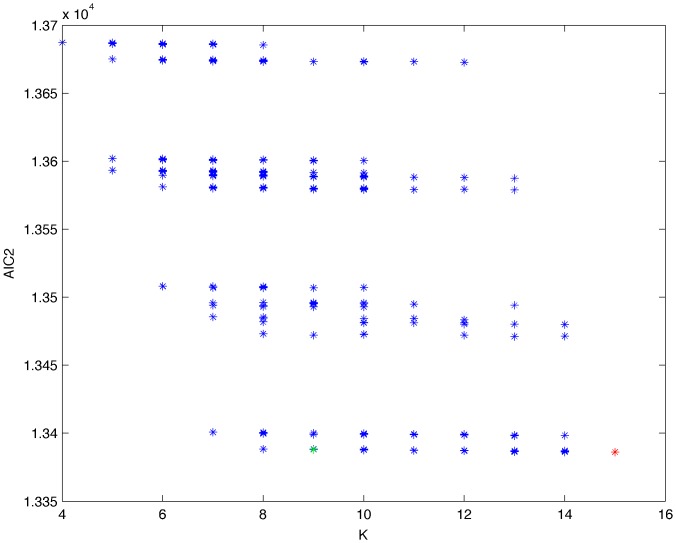
Exhaustive evaluation of AIC2 (random distribution).

**Figure 12:**
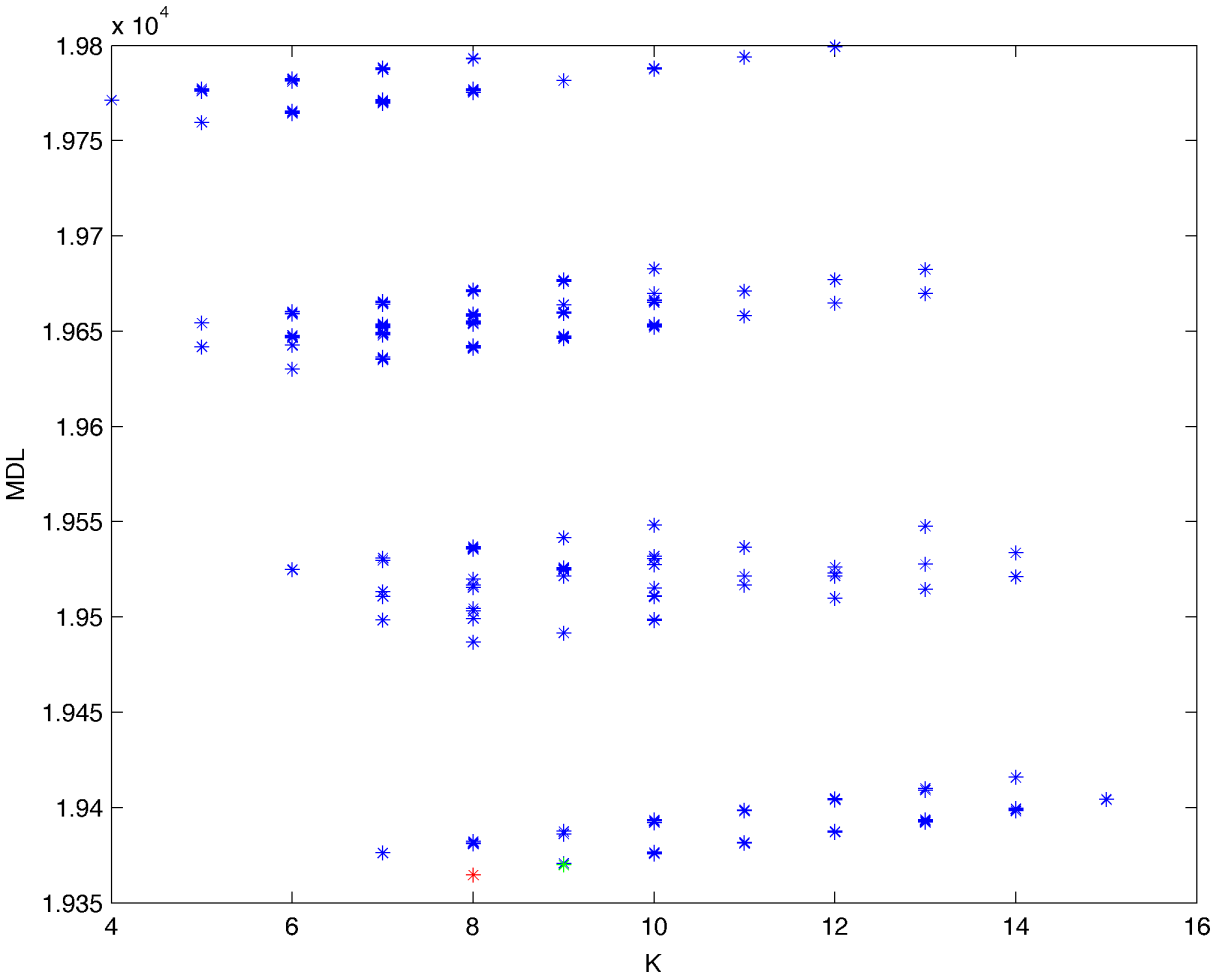
Exhaustive evaluation of MDL (random distribution).

**Figure 13:**
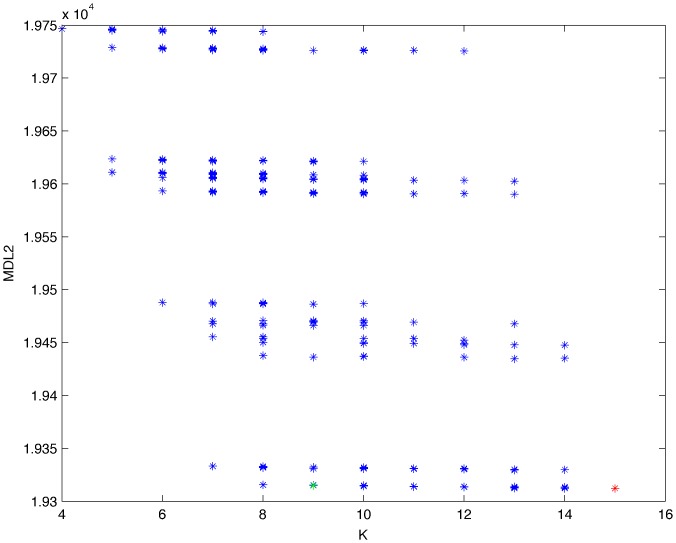
Exhaustive evaluation of MDL2 (random distribution).

**Figure 14:**
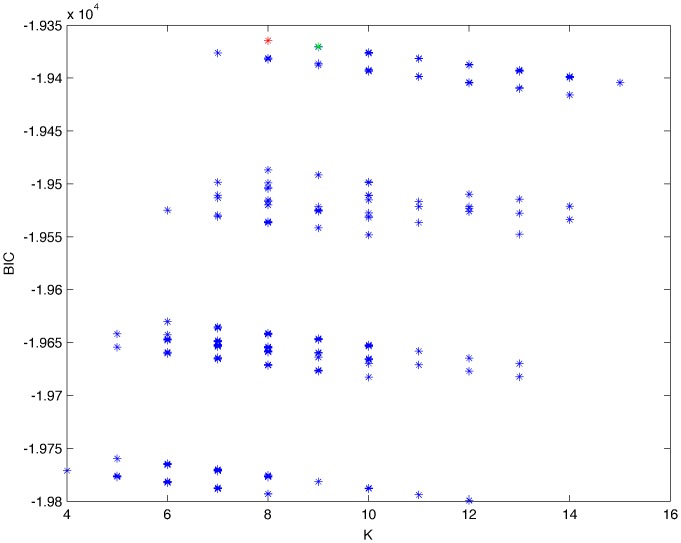
Exhaustive evaluation of BIC (random distribution).

**Figure 15:**
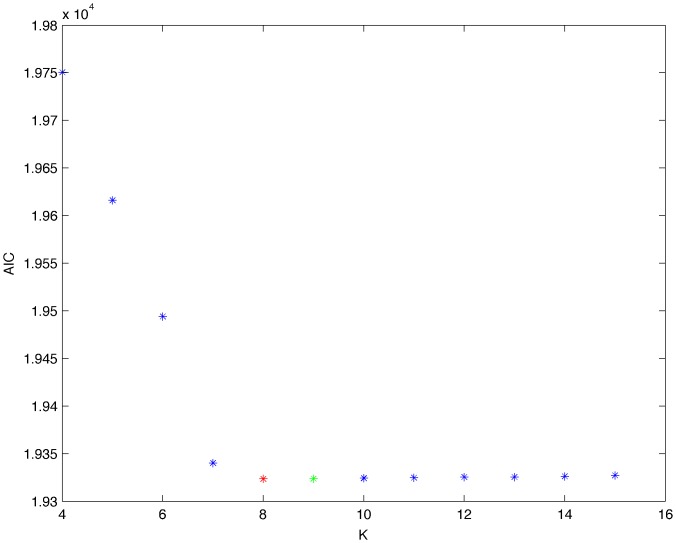
Minimum AIC values (random distribution). The red dot indicates the BN structure of Figure 20 whereas the green dot indicates the AIC value of the gold-standard network (Figure 9). The distance between these two networks = 0.000011827186444 (computed as the log2 of the ratio of gold-standard network/minimum network). A value bigger than 0 means that the minimum network has better AIC than the gold-standard.

**Figure 16:**
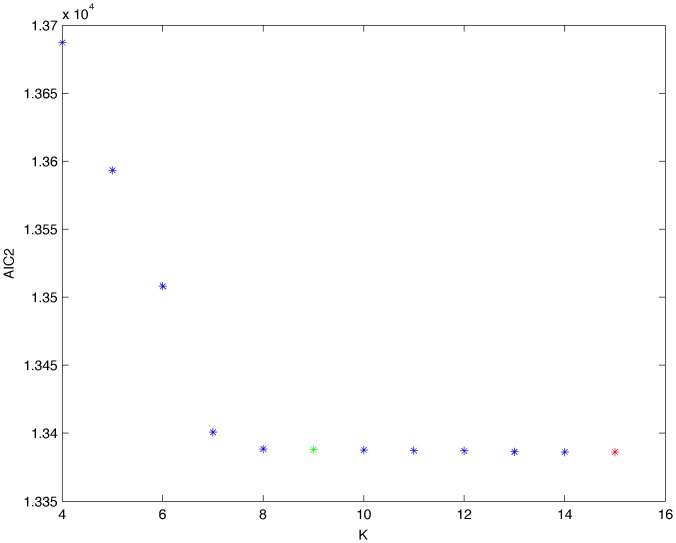
Minimum AIC2 values (random distribution). The red dot indicates the BN structure of Figure 21 whereas the green dot indicates the AIC2 value of the gold-standard network (Figure 9). The distance between these two networks = 0.000186338876083 (computed as the log2 of the ratio of gold-standard network/minimum network). A value bigger than 0 means that the minimum network has better AIC2 than the gold-standard.

**Figure 17:**
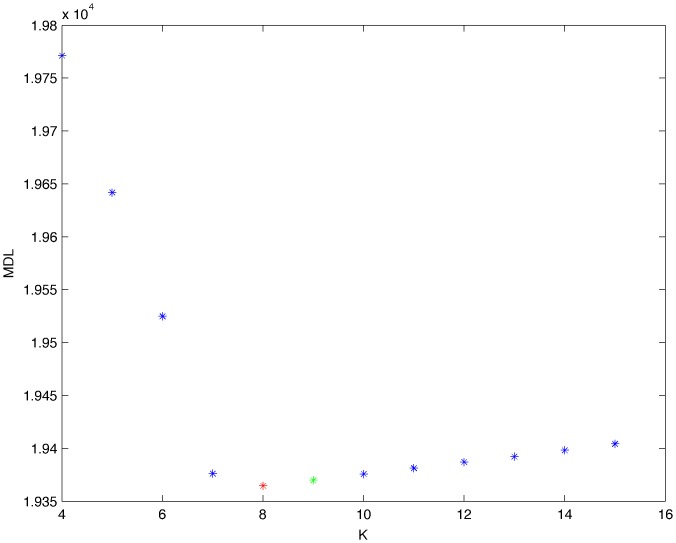
Minimum MDL values (random distribution). The red dot indicates the BN structure of Figure 20 whereas the green dot indicates the MDL value of the gold-standard network (Figure 9). The distance between these two networks = 0.00039497385352 (computed as the log2 of the ratio of gold-standard network/minimum network). A value bigger than 0 means that the minimum network has better MDL than the gold-standard.

**Figure 18:**
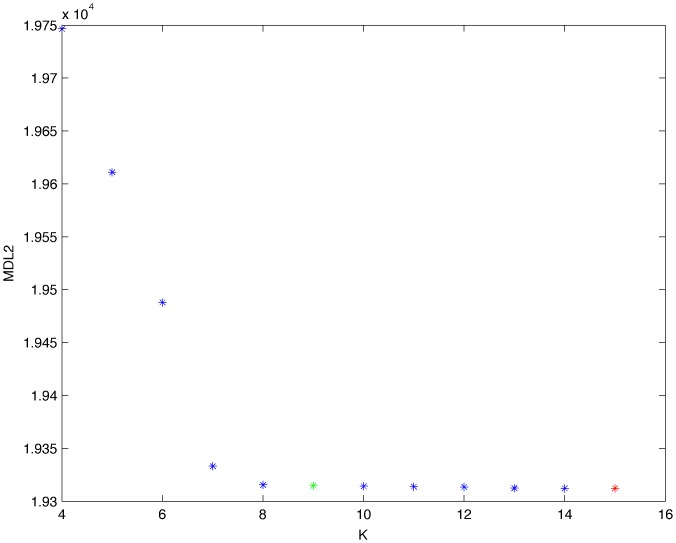
Minimum MDL2 values (random distribution). The red dot indicates the BN structure of Figure 22 whereas the green dot indicates the MDL2 value of the gold-standard network (Figure 9). The distance between these two networks = 0.00018701910455 (computed as the log_2_ of the ratio of gold-standard network/minimum network). A value bigger than 0 means that the minimum network has better MDL2 than the gold-standard.

**Figure 19:**
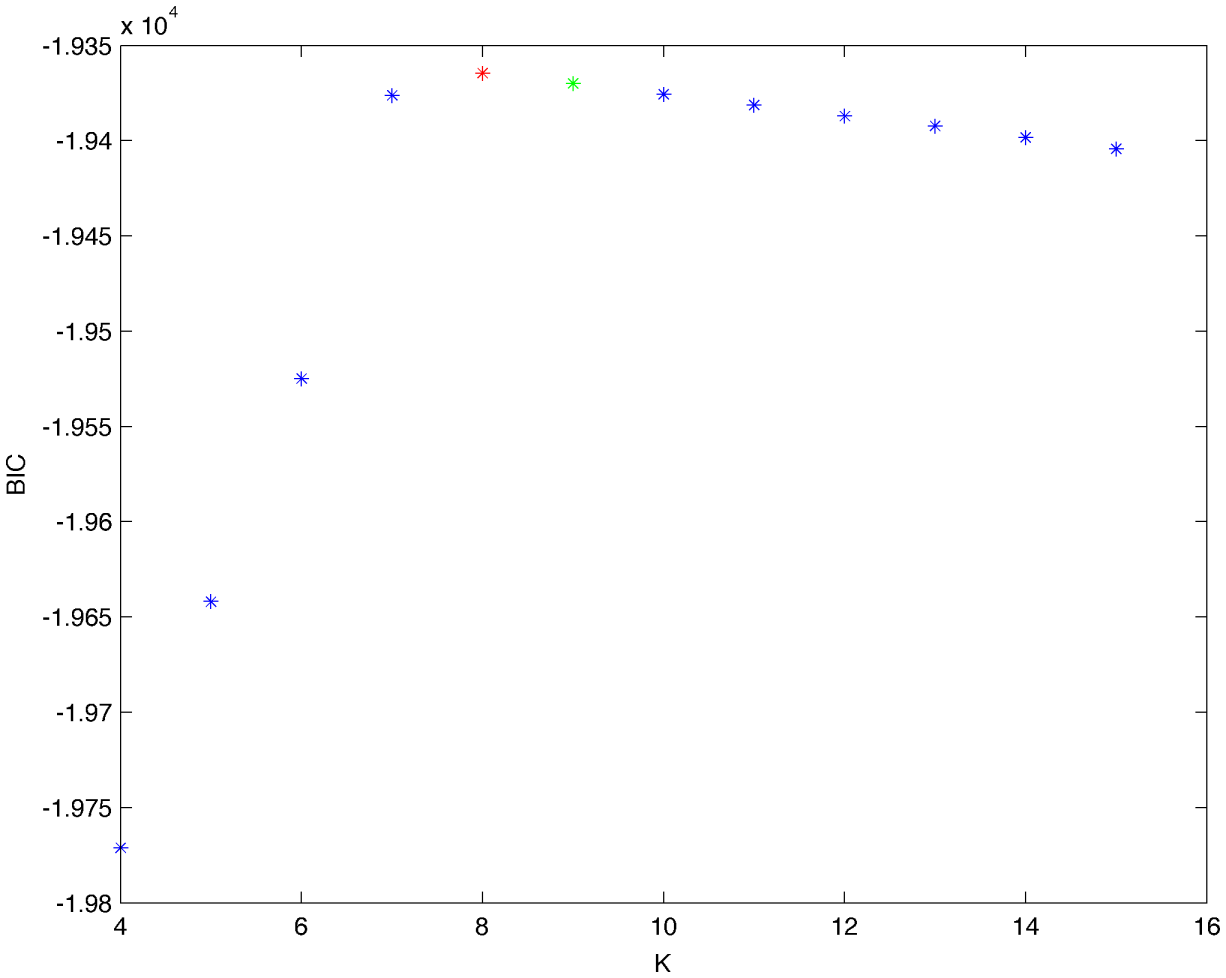
Maximum BIC values (random distribution). The red dot indicates the BN structure of Figure 20 whereas the green dot indicates the BIC value of the gold-standard network (Figure 9). The distance between these two networks = 0.00039497385352 (computed as the log2 of the ratio of gold-standard network/minimum network). A value bigger than 0 means that the minimum network has better BIC than the gold-standard.

**Figure 20:**
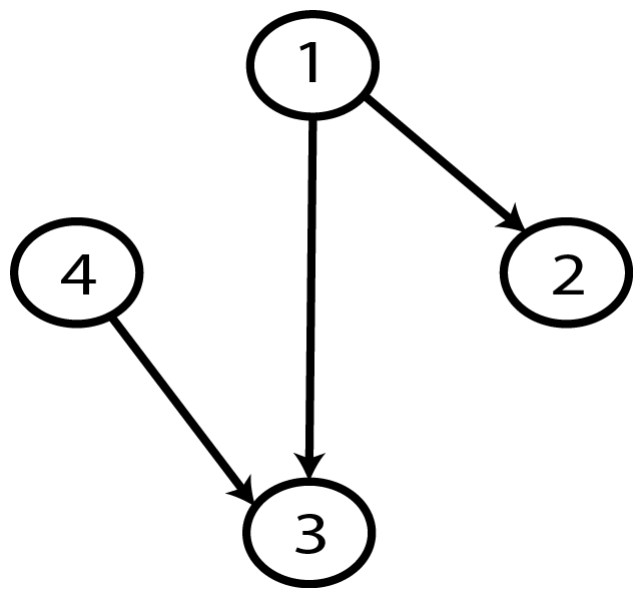
Graph with best value (AIC, MDL, BIC - random distribution).

**Figure 21:**
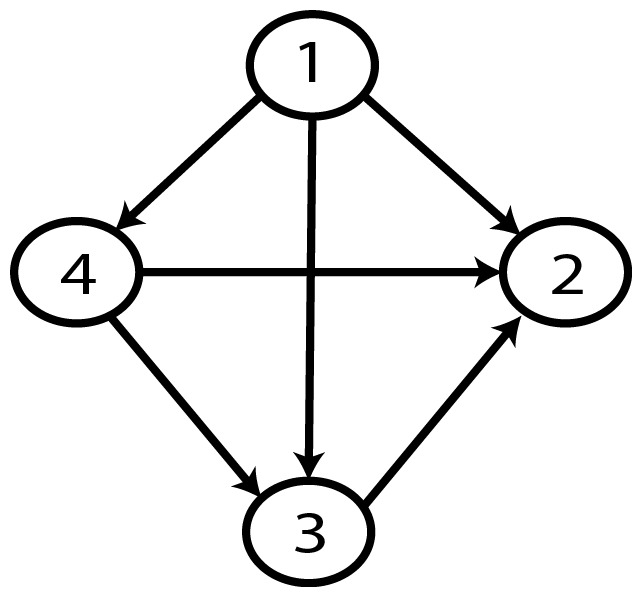
Graph with minimum AIC2 value (random distribution).

**Figure 22:**
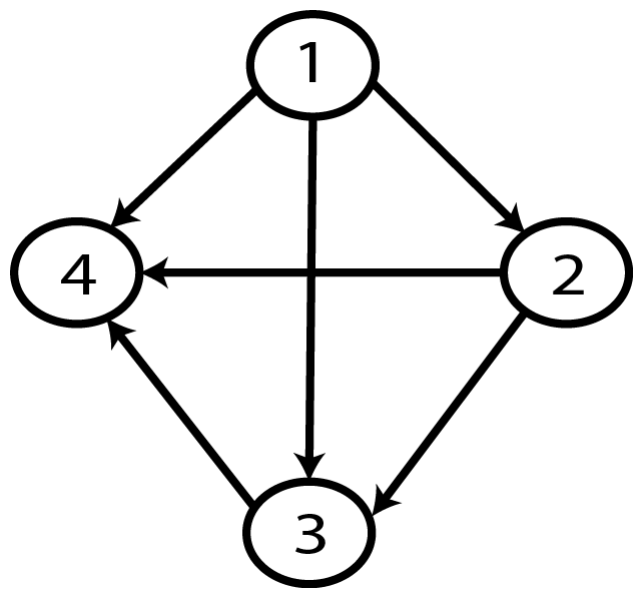
Graph with minimum MDL2 value (random distribution).

From a random gold-standard Bayesian network structure (Figure 9) and a random probability distribution, we generate 3 datasets (1000, 3000 and 5000 cases) using algorithms 1, 2 and 3 (Figures 5, 6 and 7 respectively). Then, we run algorithm 4 (Figure 8) in order to compute, for every possible BN structure, its corresponding metric value (MDL, AIC and BIC – see Equations 3 and 5–8). Finally, we plot these values (see Figures 10–14). The main goals of this experiment are, on the one hand, to check whether the traditional definition of the MDL metric (Equation 3) is enough for producing well-balanced models (in terms of complexity and accuracy) and, on the other hand, to check if such a metric is able to recover gold-standard models. Recall that some researchers (see Section ‘Introduction’) point out that the crude MDL is not complete so it should not be possible for it to come up with well-balanced models. If that is the case, other metrics such as AIC and BIC should not select well-balanced models either. That is why we also plot the values for AIC, BIC and a modified version of MDL as well [2], [6], [88]. Furthermore, regarding the second goal, other researchers claim that MDL can recover gold-standard models while others say that this metric is not specifically designed for this task. Our experiments with different sample sizes aim to check the influence of this dimension on the MDL metric itself. Here, we only show the results with 5000 cases since these are representative for all the chosen sample sizes. These results are presented in Figures 9–22. Figure 9 shows the gold-standard BN structure from which, together with a random probability distribution, the corresponding dataset is generated. Figures 10–14 show the exhaustive evaluation (blue dots) of all BN structures with the corresponding metric (AIC, AIC2, MDL, MDL2 and BIC respectively). Figures 15–19 plot only those BN structures with the minimum values for each metric and each *k*. Figure 20 shows the network with the minimum value for AIC, MDL and BIC, Figure 21 shows the network with the minimum value for AIC2 and Figure 22 shows the MDL2 minimum network.

### Experiment 2

**Figure 23:**
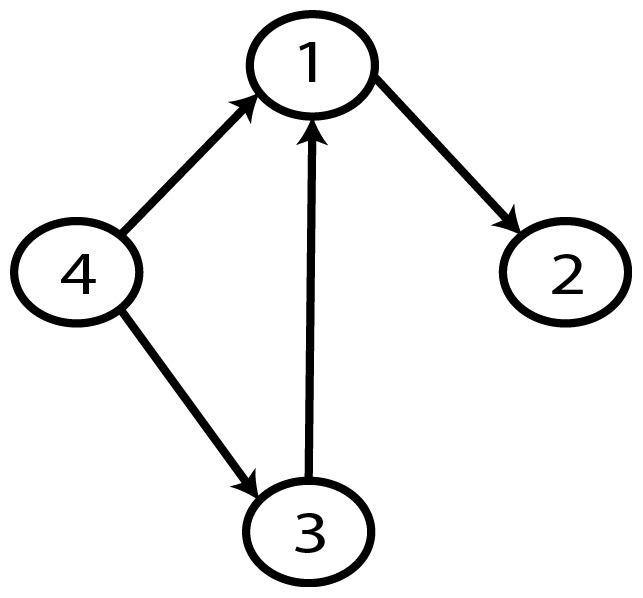
Gold-standard Network.

**Figure 24:**
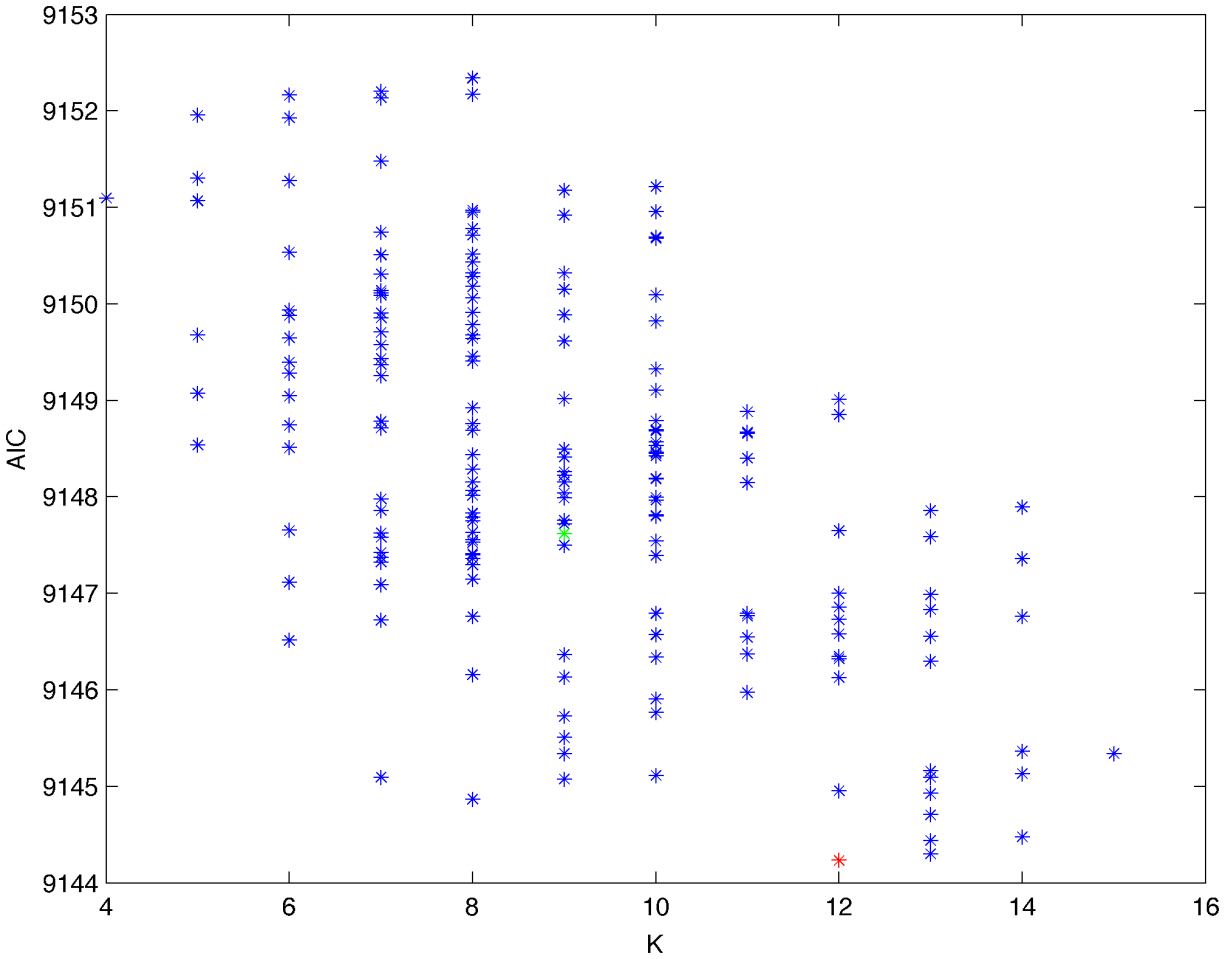
Exhaustive evaluation of AIC (low-entropy distribution).

**Figure 25:**
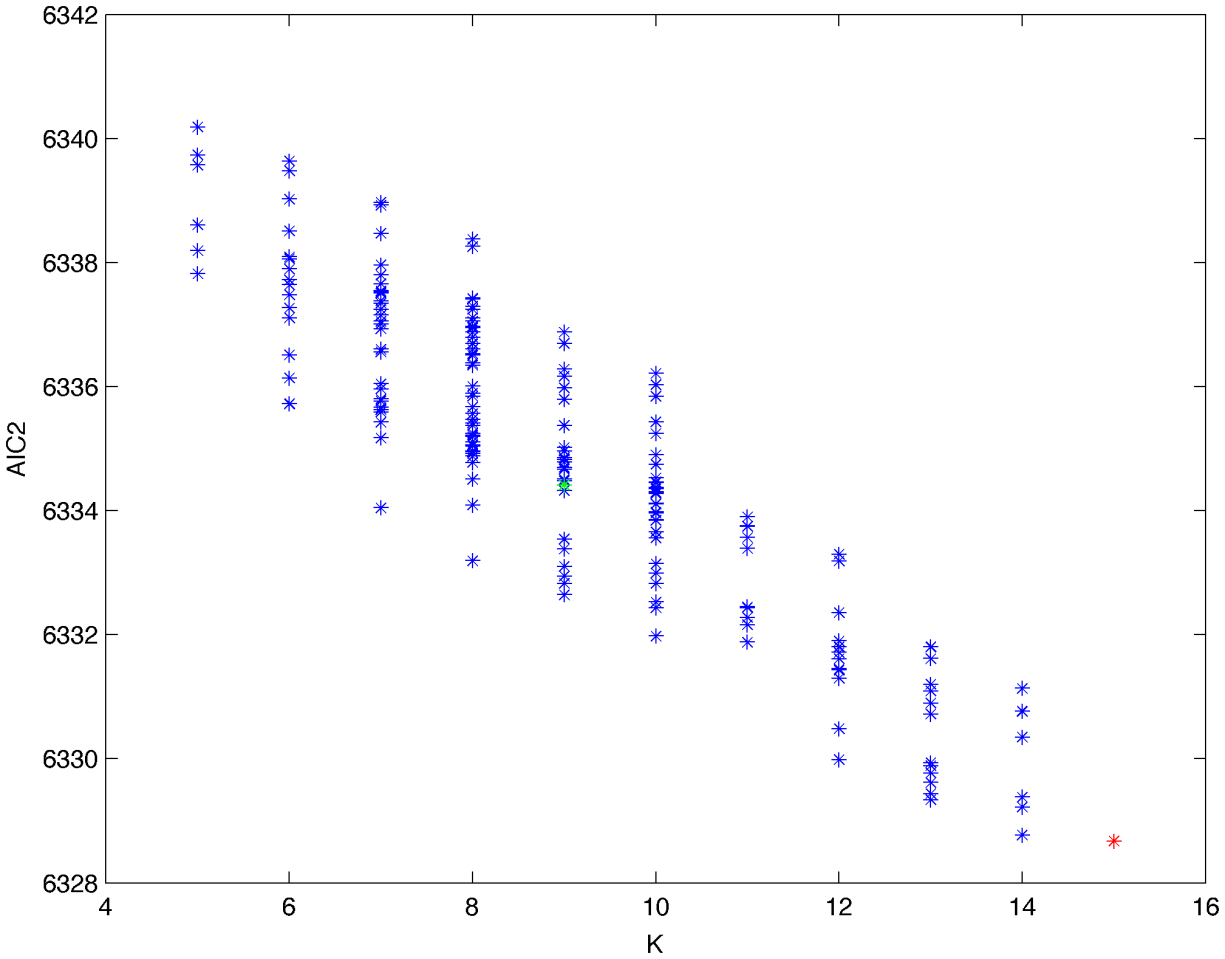
Exhaustive evaluation of AIC2 (low-entropy distribution).

**Figure 26:**
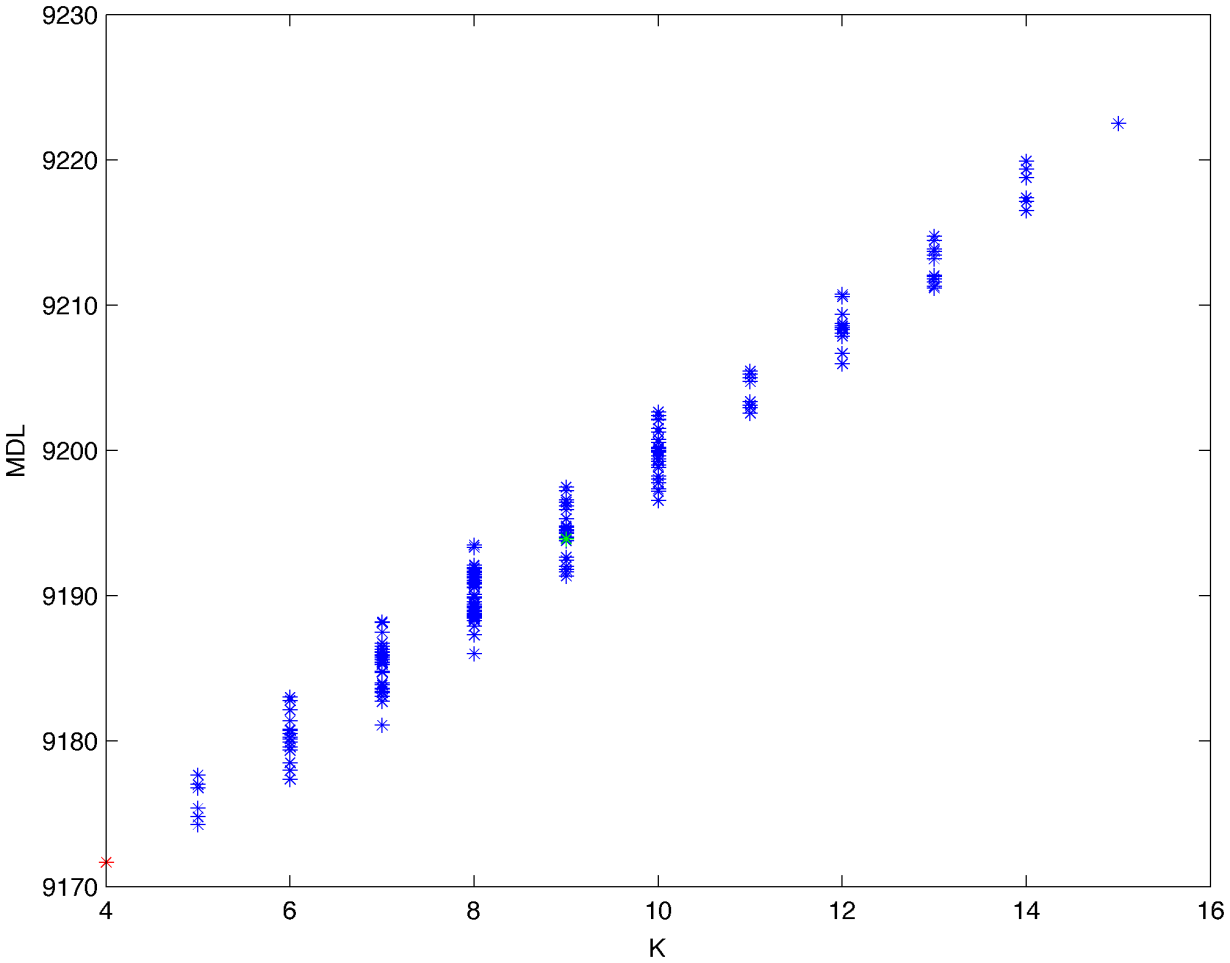
Exhaustive evaluation of MDL (low-entropy distribution).

**Figure 27:**
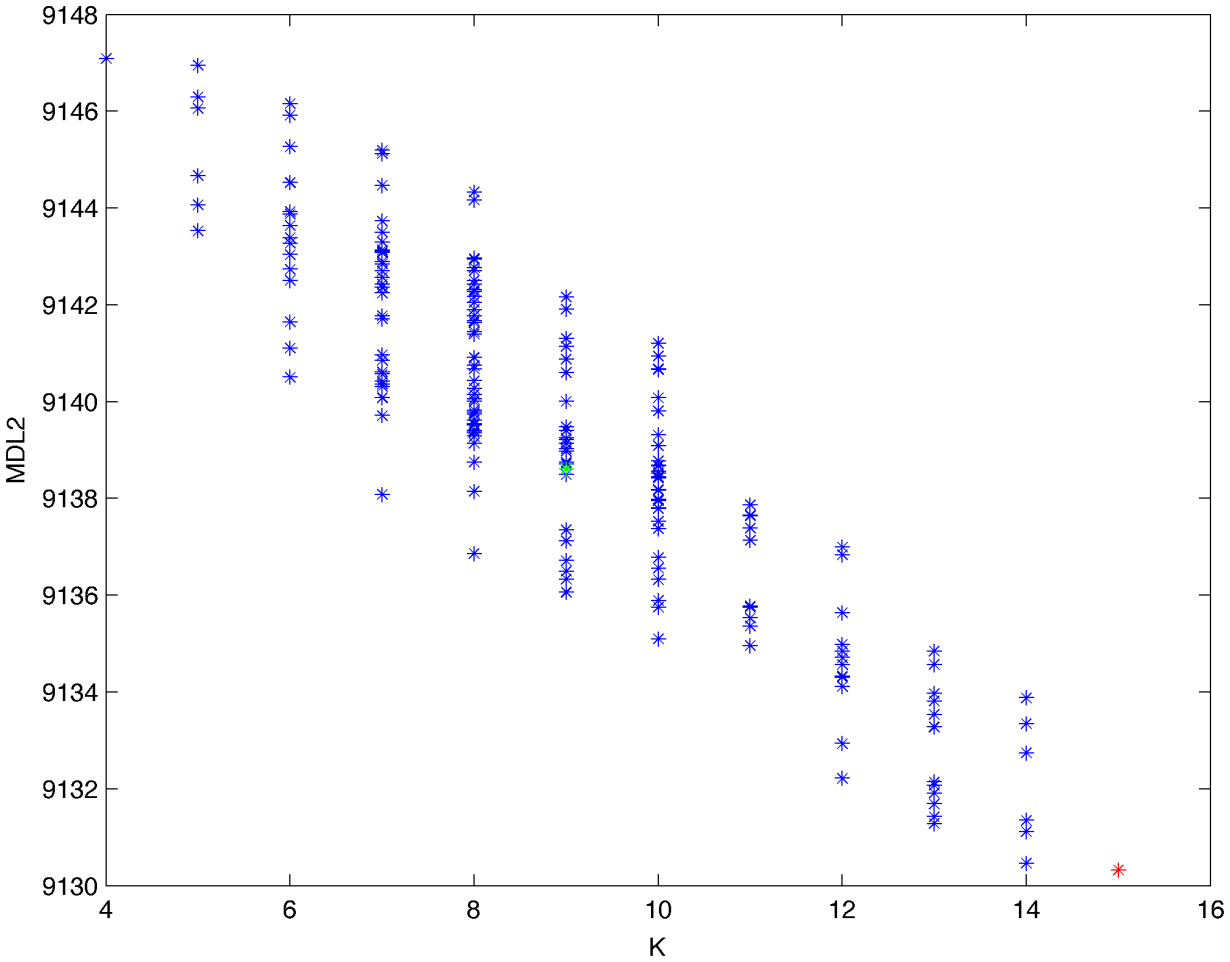
Exhaustive evaluation of MDL2 (low-entropy distribution).

**Figure 28:**
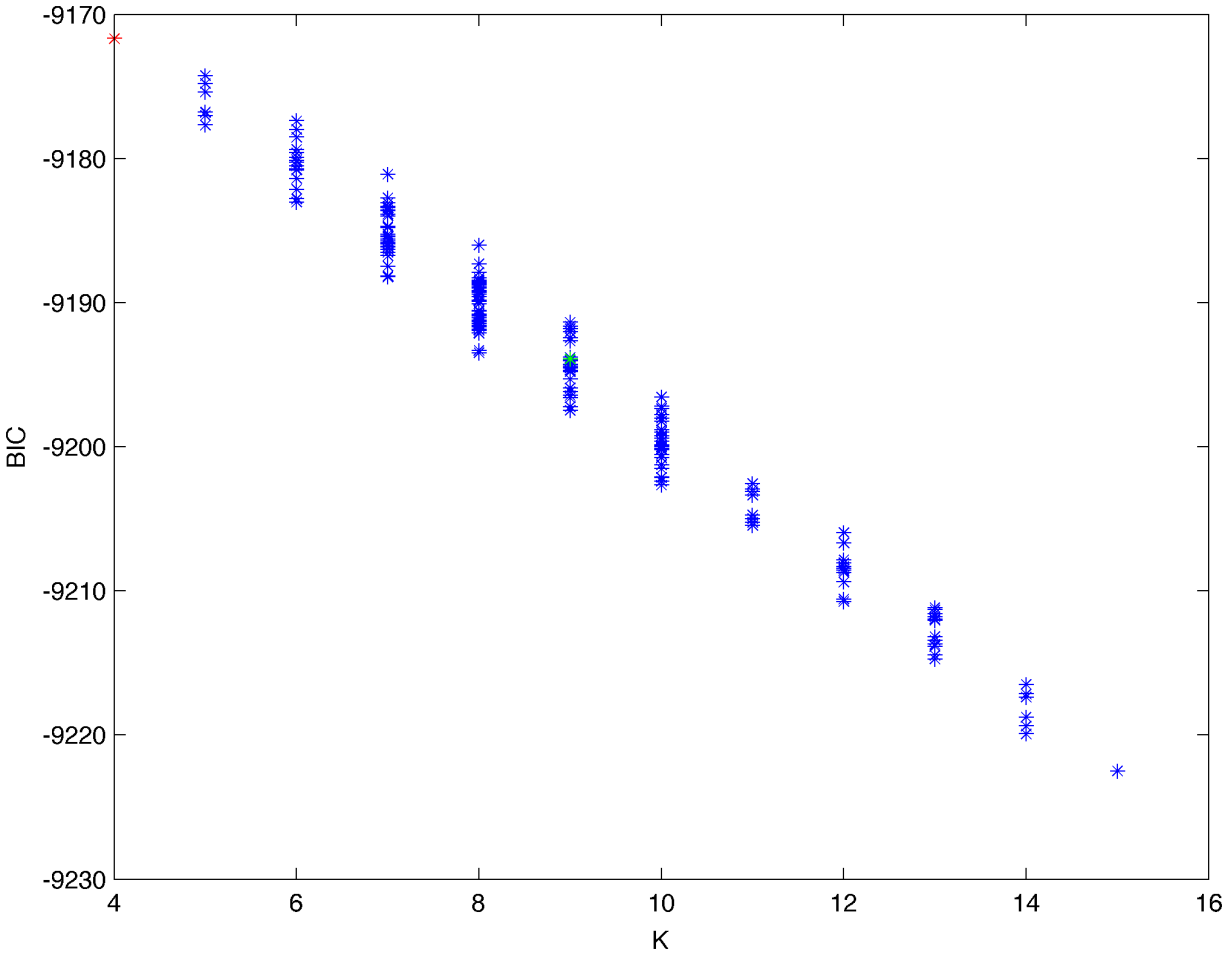
Exhaustive evaluation of BIC (low-entropy values).

**Figure 29:**
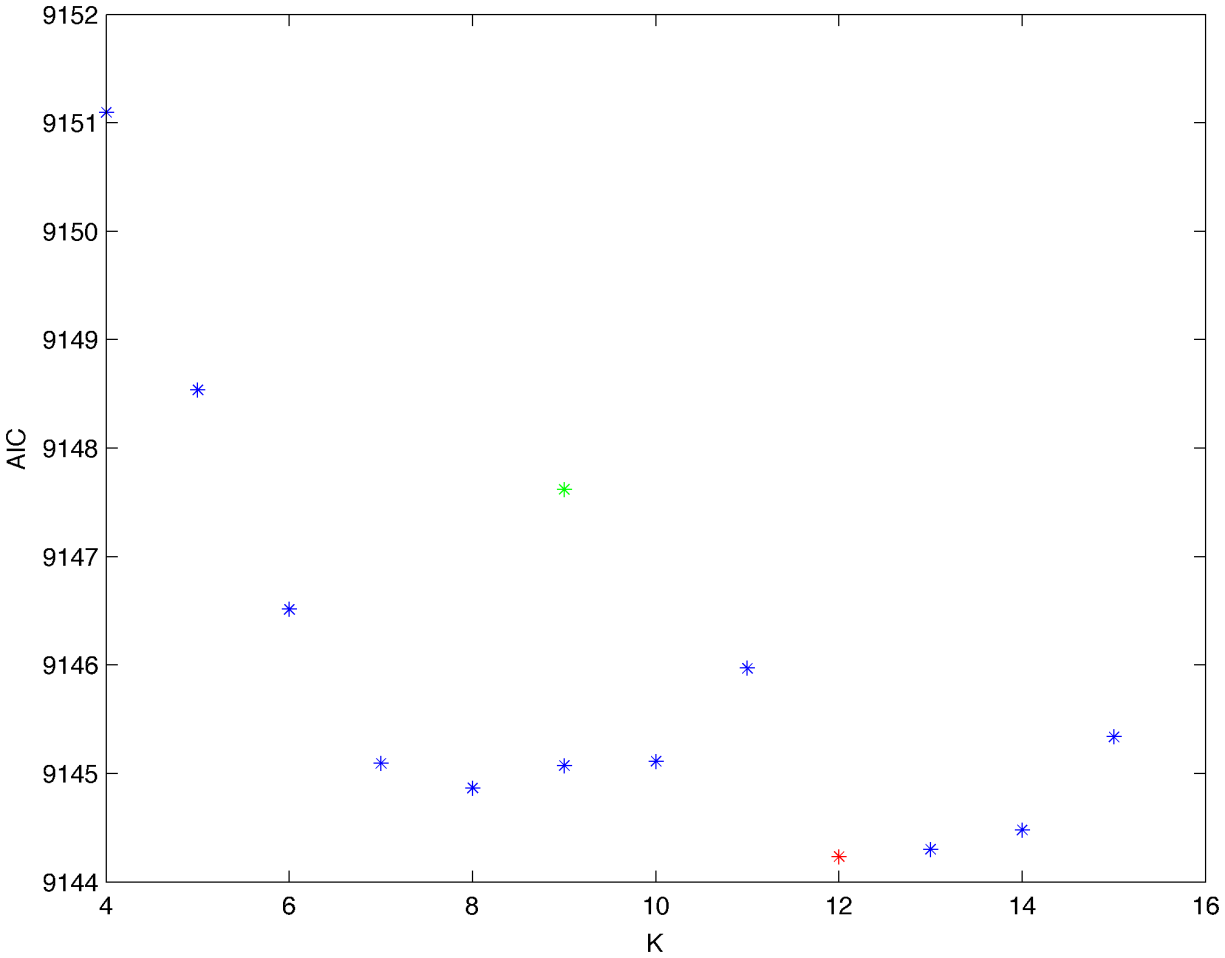
Minimum AIC values (low-entropy distribution). The red dot indicates the BN structure of Figure 34 whereas the green dot indicates the AIC value of the gold-standard network (Figure 23). The distance between these two networks = 0.00053424871665 (computed as the log_2_ of the ratio of gold-standard network/minimum network). A value bigger than 0 means that the minimum network has better AIC than the gold-standard.

**Figure 30:**
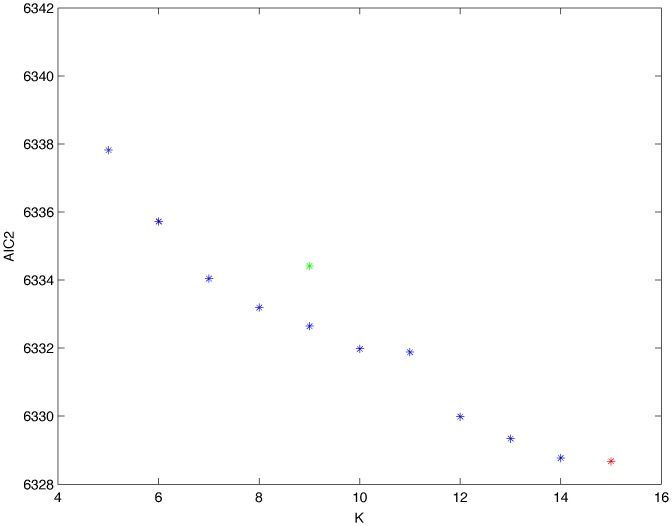
Minimum AIC2 values (low-entropy distribution). The red dot indicates the BN structure of Figure 35 whereas the green dot indicates the AIC2 value of the gold-standard network (Figure 23). The distance between these two networks = 0.001307733239164 (computed as the log_2_ of the ratio of gold-standard network/minimum network). A value bigger than 0 means that the minimum network has better AIC2 than the gold-standard.

**Figure 31:**
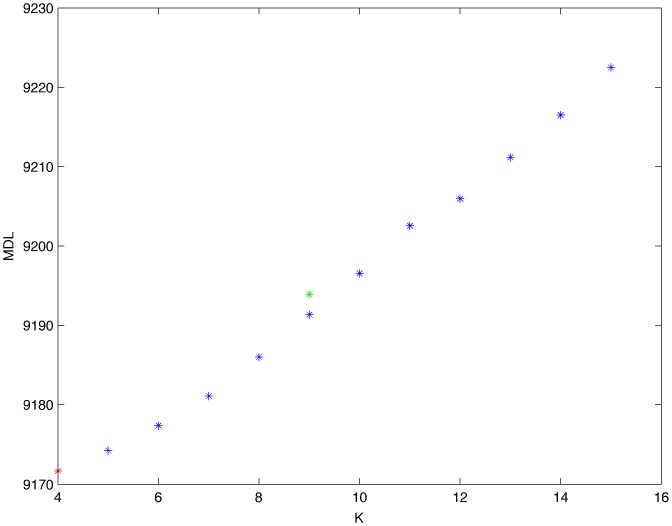
Minimum MDL values (low-entropy distribution). The red dot indicates the BN structure of Figure 36 whereas the green dot indicates the MDL value of the gold-standard network (Figure 23). The distance between these two networks = 0.003494672232915 (computed as the log_2_ of the ratio of gold-standard network/minimum network). A value bigger than 0 means that the minimum network has better MDL than the gold-standard.

**Figure 32:**
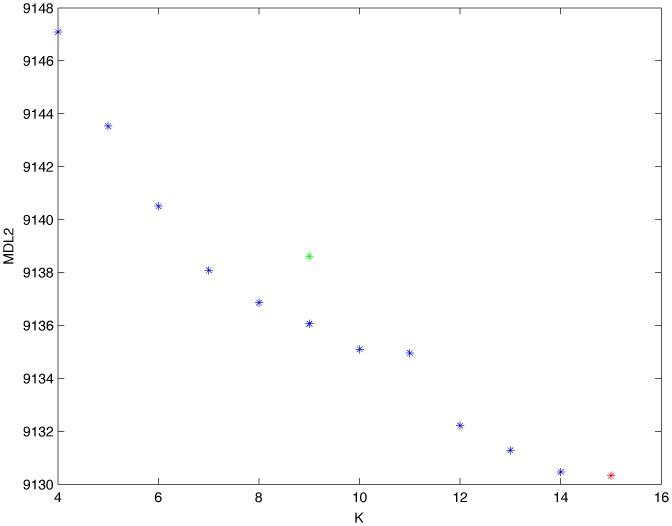
Minimum MDL2 values (low-entropy distribution). The red dot indicates the BN structure of Figure 35 whereas the green dot indicates the MDL2 value of the gold-standard network (Figure 23). The distance between these two networks = 0.001309173707777 (computed as the log_2_ of the ratio of gold-standard network/minimum network). A value bigger than 0 means that the minimum network has better MDL2 than the gold-standard.

**Figure 33:**
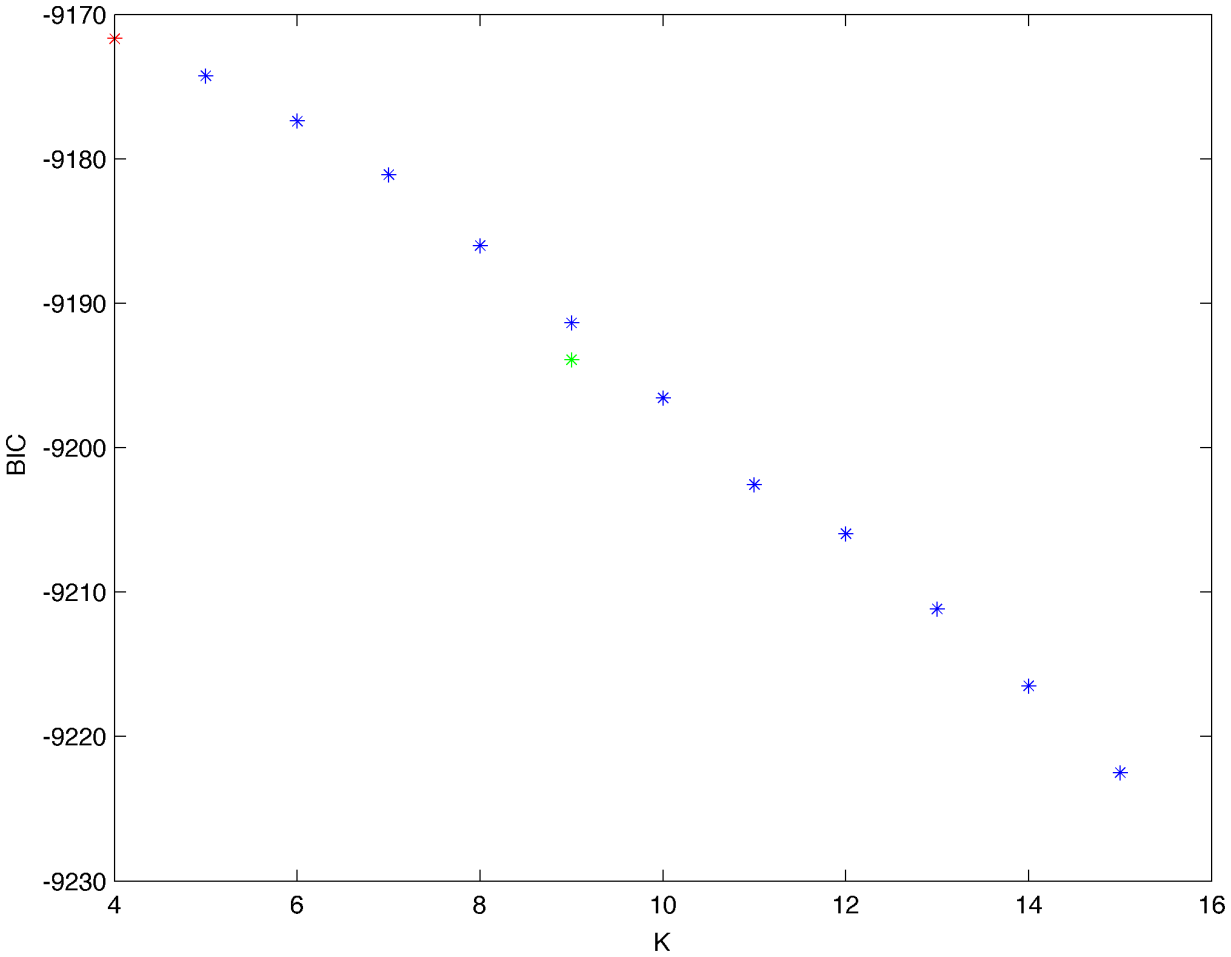
Maximum BIC values (low-entropy distribution). The red dot indicates the BN structure of Figure 36 whereas the green dot indicates the BIC value of the gold-standard network (Figure 23). The distance between these two networks = 0.003494672232915 (computed as the log_2_ of the ratio of gold-standard network/minimum network). A value bigger than 0 means that the minimum network has better BIC than the gold-standard.

**Figure 34:**
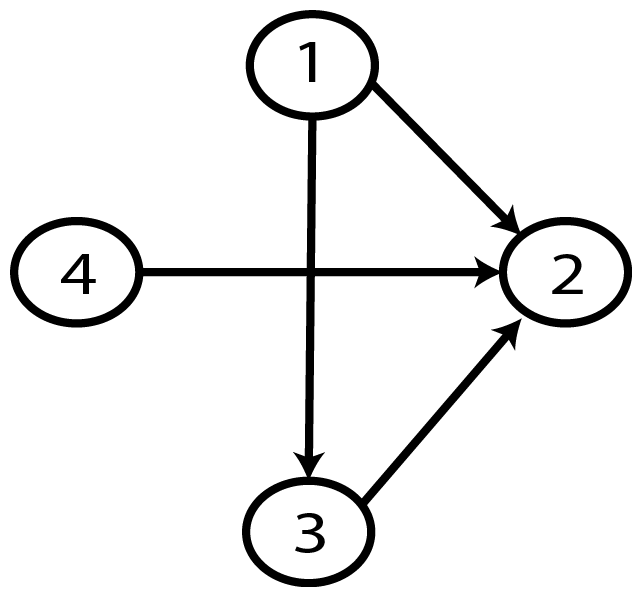
Graph with minimum AIC value.

**Figure 35:**
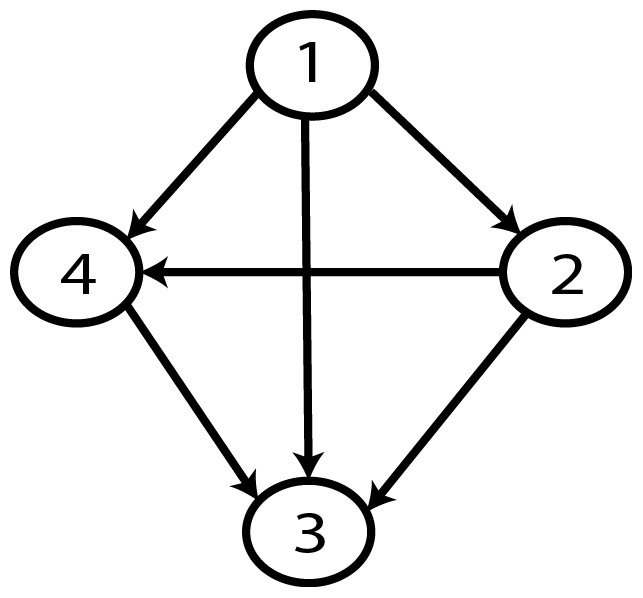
Graph with minimum AIC2 and MDL2 value.

**Figure 36:**
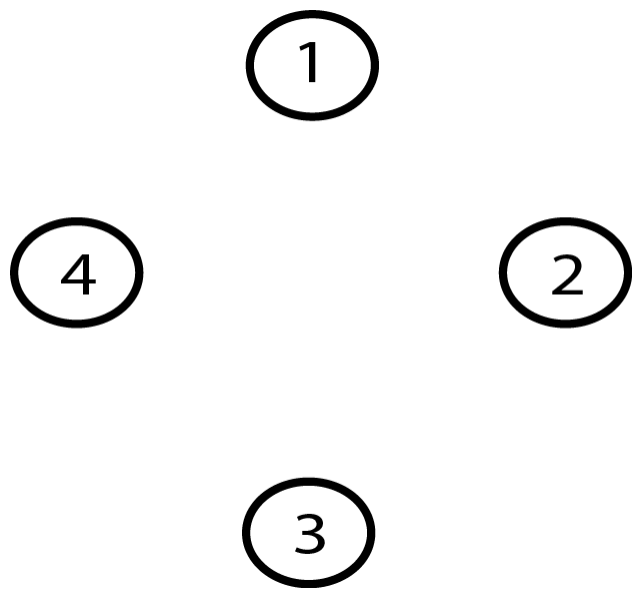
Graph with best MDL and BIC value.

From a random gold-standard Bayesian network structure (Figure 23) and a low-entropy probability distribution [6], we generate 3 datasets (1000, 3000 and 5000 cases) using algorithms 1, 2 and 3 (Figures 5, 6 and 7 respectively). According to Van Allen [6], changing the parameters to be high or low (0.9 or 0.1) tends to produce low-entropy distributions, which in turn make data have more potential to be compressed. Here, we only show experiments with distribution p = 0.1 since such a distribution is representative of different low-entropy probability distributions (0.2, 0.3, etc.). Then, we run algorithm 4 (Figure 8) in order to compute, for every possible BN structure, its corresponding metric value (MDL, AIC and BIC – see Equations 3 and 5–8). Finally, we plot these values (see Figures 24–28). The main goal of this experiment is to check whether the noise rate present in the data of Experiment 1 affects the behavior of MDL in the sense of its expected curve (Figure 4). As in Experiment 1, we evaluate the performance of the metrics in Equations 3 and 5–8. Our experiments with different sample sizes aim to check the influence of this dimension on the MDL metric itself. Here, we only show the results with 5000 cases since these are representative for all the chosen sample sizes. These results are presented in Figures 23–36. Figure 23 shows the gold-standard BN structure from which, together with a random probability distribution, the corresponding dataset is generated. Figures 24–28 show the exhaustive evaluation of all BN structures with the corresponding metric (AIC, AIC2, MDL, MDL2 and BIC respectively). Figure 29–33 plot only those BN structures with the minimum values for each metric and each *k*. Figure 34 shows the network with the minimum value for AIC; Figure 35 shows the network with the minimum value for AIC2 and MDL2 and Figure 36 shows the network with the minimum value for MDL and BIC.

## Discussion

### Experiment 1

The main goals of this experiment were, given randomly generated datasets with different sample sizes, a) to check whether the traditional definition of the MDL metric (Equation 3) was enough for producing well-balanced models (in terms of complexity and accuracy), and b) to check if such a metric was able to recover gold-standard models. To better understand the way we present the results, we give here a brief explanation on each of the figures corresponding to Experiment 1. Figure 9 presents the gold-standard network from which, together with a random probability distribution, we generate the data. Figures 10–14 show an exhaustive evaluation of each possible BN structure given by AIC, AIC2, MDL, MDL2 and BIC respectively. We plot in these figures the dimension of the model (k – X-axis) vs. the metric (Y-axis). Dots represent BN structures. Since equivalent networks have, according to these metrics, the same value, there may be more than one in each dot; i.e., dots may overlap. A red dot in each of these figures represent the network with the best metric; a green dot represents the gold-standard network so that we can visually measure the distance between these two networks. Figures 15–19 plot the minimum values of each of these metrics for every possible value for k. In fact, these figures are the result of extracting, from Figures 10–14, only the corresponding minimum values. Figure 20 shows the BN structure with the best value for AIC, MDL and BIC; Figure 21 shows the BN structure with the best value for AIC2 and Figure 22 shows the network with the best MDL2 value.

In the case of goal a), and following the theoretical characterization of MDL [17] (Figure 4), crude MDL metric seems to roughly recover its ideal behavior (see Figures 15–19). That is to say, it can be argued that crude MDL indeed finds well-balanced models in terms of accuracy and complexity, in spite of what some researchers say [2], [3]: that this version of MDL (Equation 3) is incomplete and that model selection procedures incorporating this equation will tend to choose complex models instead of simpler ones. Moreover, Grünwald [2] points out that Equation 3 (which, by the way, he calls BIC) does not work very well in practical setting when the sample size is small or moderate. In our experiments, we can see that this metric (which we call crude MDL) does indeed work well in accordance to Hastie et al. [88]: they point out that, for finite samples, BIC frequently selects models that are too simple due to its heavy penalty on complexity. Grünwald [2] also claims that AIC (Equation 5) tends to select more complex models than BIC itself because the complexity term does not depend on the sample size n. As can be observed from Figure 20, MDL, BIC and AIC all identify the same best model.

For the case of traditional formulations of AIC and MDL, although they consider that the complexity term in AIC is considerably smaller than that of MDL, our results suggest that this does not matter much since both metrics select, in general, the same minimum network. It is important to emphasize that the empirical characterization of all these metrics is one of our main contributions in this work. This characterization allows us to more easily visualize that, for instance, AIC and MDL have the same behavior, within certain limits, regardless of their respective complexity term. It can also be argued that the estimated MDL curve roughly resembles the ideal one (Figure 4).

**Figure 37:**
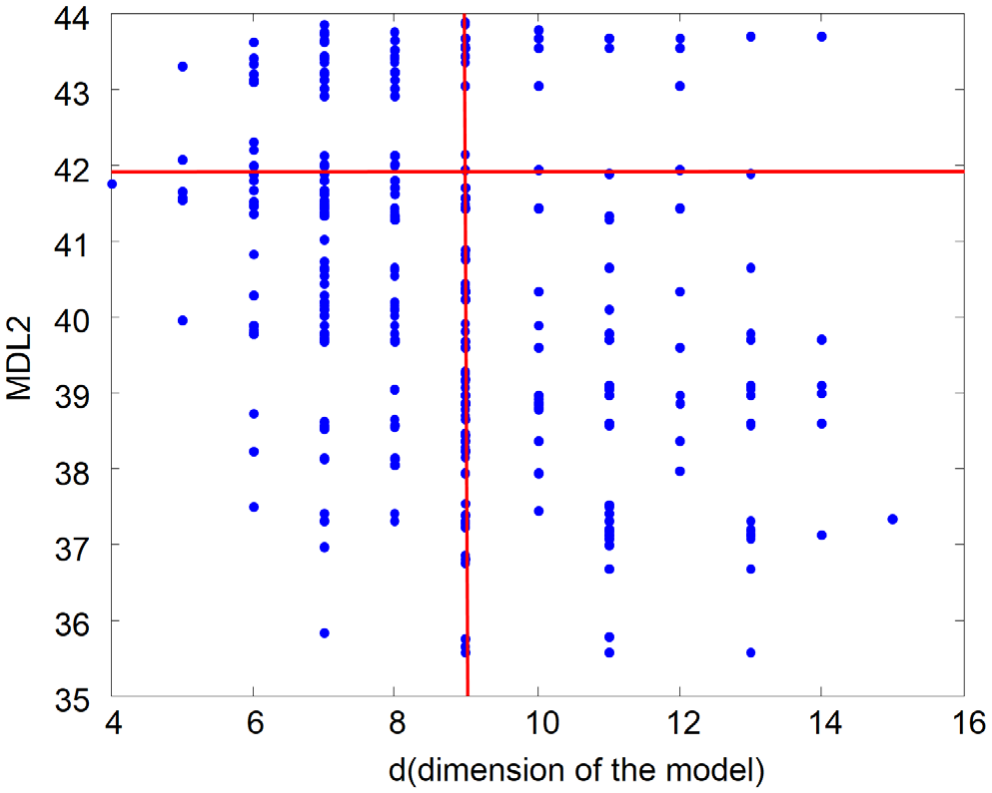
Same values for k and different values for MDL; different values for k and same values for MDL.

In the case of goal b), our results show that, most of the time, the best MDL models do not correspond to gold-standard ones, as some researchers point out [17]–[20]. In other words, as some other researchers claim, MDL is not explicitly designed for looking for the gold-standard model but for a model that well balances accuracy and complexity. In this same vein, it is worth mentioning an important case that easily escapes from observation when looking at the ideal behavior of MDL: there are at least two models that share the same dimension *k* (which, in general, is proportional to the number of arcs), yet they have different MDL score (see for instance Figure 37).

In fact, Figure 37 helps us visualize a more complete behavior of MDL: 1) there are models having a different dimension *k*, yet they have the same MDL score (see red horizontal line), and 2) there are models having the same dimension *k* but different MDL score (see red vertical line). In the first case (different complexity, same MDL), it is possible that the works reporting the suitability of MDL for recovering gold-standard networks find them since they do not perform an exhaustive search: again, their heuristic search might lead them not to find the minimal network but the gold-standard one. This means that the search procedure seeks a model horizontally. In the second case (same complexity, different MDL), it is also possible that these same works reporting the suitability of MDL for recovering gold-standard networks find such networks since they do not carry out an exhaustive search: their heuristic search might lead them not to find the minimal network but the gold-standard one. This means that the search procedure seeks a model vertically. Of course, more experimentation with such algorithms is needed so as to study more deeply their search procedures. Note that for random distributions, there are many more networks with different MDL value than their low-entropy counterparts (see for instance Figures 12 and 26).

According to Hastie et al. [88], there is no clear choice, for model selection purposes, between AIC and BIC. Remember that BIC can be considered in our experiments as equivalent to MDL. In fact, they also point out that the MDL scoring metric provides a selection criterion formally identical to the BIC score. Thus, their results match ours. It is important to mention that some researchers such as Bouckaert [17] and Hastie et al. [88] claim that, as the sample size tends to infinity, MDL and BIC can discover the gold-standard model. On the other hand, as Grünwald [2], [3] claims, the crude version of MDL is not consistent: if it were, then when there is a true distribution underlying one of the models under consideration, MDL should be able to find it provided there are enough data. Note that this does not mean that MDL is specifically designed for looking for the true distribution; rather, MDL implicitly contains a consistency sanity check: without making any distributional assumption, it should be able to identify such distribution given enough data. In our experiments, crude MDL does not find the true model but simpler models (in terms of the number of arcs).

### Experiment 2

To better understand the way we present the results, we give here a brief explanation on each of the figures corresponding to Experiment 2. Figure 23 presents the gold-standard network from which, together with a low-entropy probability distribution, we generate the data. Figures 24–28 show an exhaustive evaluation of each possible BN structure given by AIC, AIC2, MDL, MDL2 and BIC respectively. We plot in these figures the dimension of the model (k – X-axis) vs. the metric (Y-axis). Dots represent BN structures. Since equivalent networks have, according to these metrics, the same value, there may be more than one in each dot; i.e., dots may overlap. A red dot in each of these figures represent the network with the best metric; a green dot represents the gold-standard network so that we can visually measure the distance between these two networks. Figures 29–33 plot the minimum values of each of these metrics for every possible value for k. In fact, this figure is the result of extracting, from Figures 24–28, only the corresponding minimum values. Figure 34 shows the BN structure with the best value for AIC; Figure 35 shows the BN structure with the best value for AIC2 and MDL2 and Figure 36 shows the BN structure with the best MDL and BIC value. The main goal of this experiment was, given datasets with different sample sizes generated by a low-entropy distribution, to check whether the noise rate present in the data of Experiment 1 affects the behavior of MDL in the sense of its expected curve (Figure 4). In this low-entropy case, crude MDL tends to produce the empty network; i.e., the networks with no arcs (see Figure 36). We can also note that for low-entropy distributions, there are many less networks with different MDL value than their random counterparts (see Figure 26 vs. Figure 12). In the theoretical MDL graph, such a situation cannot be appreciated. Regarding the recovery of the gold-standard BN structure, it can be noted that MDL does not identify the gold-standard BN as the minimum network.

### General Considerations

Although, for the sake of brevity, we only present in the paper one experiment with a random probability distribution and sample size = 5000 and one experiment with a low-entropy distribution (p = 0.1) and sample size = 5000, we have carried out 22 more experiments with these 2 different kinds of distributions and sample size = 10000. The whole set of results can be found on the following link: http://www.lania.mx/~emezura/sites/results/. As in the experiments of the present paper, these experiments start from a random BN structure and a random/low-entropy probability distribution. Once we have both parts of the BN, we generate datasets with sample size = 10000. We thus plot each possible network in terms of the dimension of the model k (X-axis) and the metric itself (Y-axis). We also plot the minimal model for each value of k. We add in our figures the gold-standard BN structure and the minimal network so that we can visually compare their structures. We include too the data generated from the BN (structure and probability distribution) so that other systems can compare their results. Finally, we show the metric (AIC, AIC2, MDL, MDL2 or BIC) values of the gold-standard network and the minimal network and measure the distance between them (in terms of this metric). The results of these experiments support our original results: we can observe the repeatability of the latter. In fact, we have also assessed the performance of the metrics generating all possible BN structures for n = 5. These results are consistent with our original claims and can also be found on the same link.

Regarding the comparison among different procedures and ours, the codes of those procedures and/or the data used by other authors in their experiments may not be easily available. Thus, a direct comparison between them and ours is difficult. However, in order for other systems to compare their results with ours, we have made the artificial data used in our experiments available on the mentioned link.

About how the model selection process is carried out in our experiments, we should say that a strict model selection process is not performed: model selection implies not an exhaustive search but a heuristic one. In general, as seen above, an exhaustive search is prohibitive: we need to resort to heuristic procedures in order to more efficiently traverse the search space and come up with a good model that is close to the optimal one. The characterization of the MDL’s behavior presented here will help us to better understand the workings of these heuristic procedures so that we can propose some extensions for them that improve their performance. For instance, Figure 37 shows the situation where models share the same MDL but have different complexity *k* and the situation where models share the same complexity but have different MDL. This could give us an indication that a sensible heuristic should look for models diagonally instead of just vertically or horizontally. Regarding the distance between the minimum models and their corresponding gold-standard, we add Figures 15–19 for a random distribution and Figures 29–33 for a low-entropy distribution, which show, in graphical terms, such a distance. Red dots in all these figures indicate the BN structure with the best global value whereas green dots indicate the value of the gold-standard networks. This visualization may be also useful in the design of a heuristic procedure.

## Conclusions and Future Work

In this work, we have thoroughly evaluated the graphical performance of crude MDL as a metric for BN model selection: this is the main contribution of the paper. We argue that without such graphical performance MDL’s behavior is hard to imagine. Figures showing this behavior tell us a more complete and clearer story: crude MDL is inconsistent in the sense of its incapability for recovering gold-standard BN. Moreover, these figures also show that, with even few variables, the search procedure will have a hard time to come up with the minimum network. We indeed generated every possible network (for the case of n = 4) and measure, for each one of them, its corresponding metric (AIC, AIC2, MDL, MDL2 and BIC). Since, in general, it is practically impossible to search over the whole BN structure space, a heuristic procedure must be used. However, with this kind of procedure it is not, strictly speaking, possible to find the best global model. On the other hand, as can be noted, the experiments presented here involve an exhaustive search, thus making it possible to identify this best global model. The connection between a heuristic search and an exhaustive one, from the point of view of our experiments, is that the results of such an exhaustive characterization may allow us to better understand the behavior of heuristic procedures since we can easily compare the model produced by the latter and the minimal model identified by the former. In doing so, we might track the steps a specific heuristic algorithm follows to come up with the final model: this in turn may allow us to design an extension so that this algorithm improves and generalizes its performance to problems involving more than 4 variables. In sum, as a future work, we will try to design different heuristics in order to more efficiently find networks close to the best ones, thus avoiding overfitting (networks with many arcs). As can be seen then, no novel selection method is proposed since this is not the goal of the paper. Furthermore, no real-world data have been considered in the experiments carried out here for such an analysis would not allow, by definition, to know a priori the gold-standard network and thus to assess the performance of crude MDL as a metric capable of recovering these gold-standard models. Even if we could know a priori such models, real-world data usually contain a number of variables (more than 6) that would render the exhaustive computation of crude MDL for each possible BN infeasible. Our findings may be applied to real systems in the sense of making one fully aware that the minimum crude MDL network will not, in general, be the gold-standard BN and that the selection of a good model depends not only upon this metric but also upon other dimensions (see below).

According to the previous results in the study of this metric (see Section ‘Related work’), we can identify 2 schools of thought: 1) those who claim that the traditional formulation of MDL is not complete and hence needs to be refined, for it cannot select well-balanced models (in terms of accuracy and complexity); and 2) those who claim that this traditional definition is enough for finding the gold-standard model, which in our case is a Bayesian network. Our results can be situated somewhat in the middle: they suggest that the traditional formulation of MDL does indeed choose well-balanced models (in the sense of recovering the ideal graphical behavior of MDL) but that this formulation is not consistent (in the sense of Grünwald [2]): given enough data, it does not recover the gold-standard model.

These results have led us to detect 4 probable sources for the differences among different schools: 1) the metric itself, 2) the search procedure, 3) the noise rate and 4) the sample size.

In the case of 1), we still have to test the refined version of MDL to check whether it works better than its traditional counterpart in the sense of consistency: if we know for sure that a specific probability distribution actually generated the data, MDL should be able to find it [2]. As can be seen from our results, the crude version of MDL is not able to find such distribution: this may suggest that this version is not completely consistent. Thus, we have to evaluate whether the refined version of MDL is more consistent than its traditional counterpart. This consistency test is left as future work. Recall that such a metric extends its crude version in the sense of the complexity term: it also takes into account the functional form of the model (i.e., its geometrical/structural properties) [2]. From this extension, we can infer that this functional form more accurately reflects the complexity of the model. We propose then the incorporation of Equation 4 for the same set of experiments presented here.

In the case of 2), our results suggest that, since the related works presented in Section ‘Related work’ do not carry out an exhaustive search, the gold-standard network often reflects a good tradeoff between accuracy and complexity but this does not necessarily mean that such a network is the one with the best MDL score (in the graphical sense given by Bouckaert [17]). Thus, it can be argued that the responsible for coming up with this gold-standard model is the search procedure. Of course, it is necessary, in order to reduce the uncertainty of this assertion, to carry out more tests regarding the nature of the search mechanism. This is also left as future work. Given our results, we may propose a search procedure that works diagonally instead of only vertically or horizontally (see Figure 37). If our search procedure only seeks vertically or horizontally, it can get trapped in the problems mentioned in Section ‘Discussion’: it may find models with the same complexity and different MDL or models with the same MDL but different complexity respectively. We would like to have a search procedure that looks simultaneously for models with better *k* and MDL.

In the case of 3), the investigation by Kearns et al. [4] shows that while more noise is added, MDL needs more data to reduce its generalization error. Although their results have to do more with the classification performance of MDL, they are related to ours in the sense of the power of this metric for selecting a well-balanced model that, it can be argued, is useful for classification purposes. Their finding gives us a clue regarding the possibility of a well-balanced model (perhaps the gold-standard one - depending on the search procedure) to be recovered as long as there are enough data and not much noise. In other words, MDL might not select a good model in the presence of noise, even when the sample size is large. Our results show that, when using a random distribution, the recovered MDL graph closely resembles the ideal one. On the other hand, when a low-entropy distribution is present, the recovered MDL curve only slightly resembles the ideal one.

In the case of 4), our findings suggest that when a sample size limit is reached, the results do not considerably change. However, we need to carry out more experimentation in the sense of checking the consistency of the definition of MDL (both crude and refined) regarding the sample size; i.e., MDL should be able to identify the true distribution given enough data [2] and not much noise [4]. This experimentation is left as future work as well.

We also plan to implement and compare different search algorithms in order to assess the influence of such a dimension in the behavior of MDL. Recall that the search algorithm is an important ingredient for finding simpler models over complex ones. We plan to plot the refined version of MDL and to check its consistency as well. Furthermore, we plan to test the influence of the sample size in the behavior of MDL. Other tests we are planning to carry out as a future work have to do with a deeper assessment of the performance of all the metrics with 6 nodes.

Finally, a deeper comparison among the different metrics presented here (in the sense of the four dimensions discussed above) is also left as a future work.

## References

[1] Grünwald P (2000) Model selection based on Minimum Description Length. J Math Psychol 44 (1): 133–152. 10.1006/jmps.1999.1280 10733861

[2] Grünwald P (2007) The Minimum Description Length principle. MIT Press. 703 p.

[3] Grunwald P, Myung IJ, Pitt MA, eds. (2005) Advances in Minimum Description Length: theory and applications. MIT Press. 452 p.

[4] Kearns M, Mansour Y, Ng AY, Ron D (1997) An experimental and theoretical comparison of model selection methods. Mach Learn 27 (1): 7–50.

[5] Myung IJ (2000) The importance of complexity in model selection. J Math Psychol 44 (1): 190–204. 10.1006/jmps.1999.1283 10733864

[6] Van Allen T, Greiner R (2000) Model selection criteria for learning belief nets: an empirical comparison. Proc Int Conf Mach Learn 17: 1047–1054.

[7] Zucchini W (2000) An introduction to model selection. J Math Psychol 44 (1): 41–61. 10.1006/jmps.1999.1276 10733857

[8] Bozdogan H (2000) Akaike’s information criterion and recent developments in information complexity. J Math Psychol 44 (1): 62–91. 10.1006/jmps.1999.1277 10733858

[9] Pearl J (1988) Probabilistic reasoning in intelligent systems: networks of plausible inference. San Mateo, California: Morgan Kaufmann. 552 p.

[10] Wasserman L (2000) Bayesian model selection and model averaging. J Math Psychol 44 (1): 92–107. 10.1006/jmps.1999.1278 10733859

[11] Cooper GF (1999) An overview of the representation and discovery of causal relationships using Bayesian networks. In: Glymour C and Cooper GF, editors. Computation, causation & discovery. AAAI Press/MIT Press. 3–62.

[12] Geiger D, Heckerman D, Meek C (1998) Asymptotic model selection for directed networks with hidden variables. Learning in graphical models, NATO ASI series 89: 461–477.

[13] Heckerman D (1998) A tutorial on learning with Bayesian networks. Learning in graphical models, NATO ASI series 89: 301–354.

[14] Friedman JH (1997) On bias, variance, 0/1-loss, and the curse of dimensionality. Data Min Knowl Discov 1 (1): 55–77.

[15] Geman S, Bienenstock E, Doursat R (1992) Neural Networks and the bias/variance dilemma. Neural Comput 4 (1): 1–58.

[16] Hastie T, Tibshirani R, Friedman J (2001) The elements of statistical learning. New York: Springer. 533 p.

[17] Bouckaert RR (1993) Probabilistic network construction using the Minimum Description Length principle. In: Clarke M, Kruse R and Moral S, editors. Symbolic and quantitative approaches to reasoning and uncertainty. Springer-Verlag. 41–48.

[18] Lam W, Bacchus F (1994) Learning Bayesian belief networks: an approach based on the MDL principle. Comput Intell 10 (3): 269–293.

[19] Suzuki J (1996) Learning Bayesian belief networks based on the Minimum Description Length principle: an efficient algorithm using the B & B technique. Proc Int Conf Mach Learn 13: 462–470.

[20] Suzuki J (1999) Learning Bayesian belief networks based on the Minimum Description Length Principle: basic properties. IEICE transactions on fundamentals of electronics, communications and computer science E82-A (10): 2237–2245.

[21] Robinson RW (1977) Counting unlabeled acyclic digraphs. Combinatorial mathematics V, Lecture notes in mathematics 622: 28–43.

[22] Chickering DM, Heckerman D, Meek C (1997) A Bayesian approach to learning Bayesian networks with local structure. Uncertain Artif Intell 13: 80–89.

[23] Cooper GF, Herskovits E (1992) A Bayesian method for the induction of probabilistic networks from data. Mach Learn 9: 309–347.

[24] Friedman N, Geiger D, Goldszmidt M (1997) Bayesian network classifiers. Mach Learn 29 (2–3): 131–163.

[25] Glymour C, Cooper GF, eds. (1999) Computation, causation & discovery. AAAI Press/MIT Press. 552 p.

[26] Heckerman D, Geiger D, Chickering DM (1995) Learning Bayesian networks: the combination of knowledge and statistical data. Mach Learn 20 (3): 197–243.

[27] Jordan MI, ed. (1998) Learning in graphical models. Dordecht, The Netherlands: Kluwer Academic Publishers. 634 p.

[28] Neapolitan RE (1990) Probabilistic reasoning in expert systems: theory and algorithms. New York: John Wiley & Sons, Inc. 433 p.

[29] Pearl J (2000) Causality: models, reasoning and inference. New York: Cambridge University Press. 384 p.

[30] Spirtes P, Glymour C, Scheines R (1993) Causation, prediction and search. New York: Springer-Verlag. 526 p.

[31] Spirtes P, Meek C (1995) Learning Bayesian networks with discrete variables from data. KDD 1: 294–299.

[32] Whittaker J (1990) Graphical Models in Applied Mathematical Multivariate Statistics. New York: John Wiley & Sons. 448 p.

[33] Friedman N, Goldszmidt M (1998) Learning Bayesian networks from data. Proc Conf AAAI Artif Intell 15.

[34] Buntine W (1996) A guide to the literature on learning probabilistic networks from data. IEEE Trans Knowl Data Eng 8 (2): 195–210.

[35] Diez FJ, Mira J, Iturralde E, Zubillaga S (1997) DIAVAL, a Bayesian expert system for echocardiography. Artif Intell Med 10 (1): 59–73. 10.1016/s0933-3657(97)00384-9 9177816

[36] Chickering DM (1996) Learning Bayesian Networks is NP-Complete. Learning from data, Lecture notes in statistics 112: 121–130.

[37] Russell S, Norvig P (2002) Artificial intelligence: a modern approach. Prentice Hall. 1179 p.

[38] Grossman D, Domingos P (2004) Learning Bayesian network classifiers by maximizing conditional likelihood. Proc Int Conf Mach Learn 21: 46.

[39] Kelner R, Lerner B (2012) Learning Bayesian network classifiers by risk minimization. Int J Approx Reason 53: 248–272.

[40] Acid S, de Campos LM, Fernández M (2013) Score-based methods for learning Markov boundaries by searching in constrained spaces. Data Min Knowl Discov 26 (1): 174–212.

[41] Chow CK, Liu CN (1968) Approximating discrete probability distributions with dependence trees. IEEE Trans Inf Theory 14 (3): 462–467.

[42] Friedman N, Goldszmidt M (1996) Discretizing continuous attributes while learning Bayesian networks. Proc Int Conf Mach Learn 13: 157–165.

[43] Cheng J, Greiner R (1999) Comparing Bayesian network classifiers. Uncertain Artif Intell 15: 101–108.

[44] Kontkanen P, Silander T, Myllymaki P, Tirri H (1999) On supervised selection of Bayesian networks. Uncertain Artif Intell 15: 334–342.

[45] Kontkanen P, Myllymaki P, Silander T, Tirri H, Grünwald P (2000) On predictive distributions and Bayesian networks. Stat Comput 10 (1): 39–54.

[46] Kleiner A, Sharp B (2000) A new algorithm for learning Bayesian classifiers from data. Artificial Intelligence and Soft Computing: 191–197.

[47] Clarke EJ, Barton BA (2000) Entropy and MDL discretization of continuous variables for Bayesian belief networks. International Journal of Intelligent Systems 15 (1): 61–92.

[48] Madden MG (2002) A new Bayesian network structure for classification tasks. Artificial intelligence and cognitive science, Lecture notes in computer science 2464: 203–208.

[49] Wong ML, Lee SY, Leung KS (2002) A hybrid data mining approach to discover Bayesian networks using evolutionary programming. GECCO 2: 214–222.

[50] Wong ML, Lee SY, Leung KS (2002) A hybrid approach to discover Bayesian networks from databases using evolutionary programming. Proc IEEE Int Conf Data Min 2002: 498–505.

[51] Madden M (2003) The performance of Bayesian network classifiers constructed using different techniques. Proceedings of European conference on machine learning, workshop on probabilistic graphical models for classification 14: 59–70.

[52] Santos E, Hussein A (2004) Case-based Bayesian network classifiers. FLAIRS conference 2004: 538–543.

[53] Roos R, Wettig H, Grünwald P, Myllynäki P, Tirri H (2005) On discriminative Bayesian network classifiers and logistic regression. Mach Learn 59 (3): 267–296.

[54] Castillo G, Gama J (2005) Bias management of Bayesian network classifiers. Discovery science, Lecture notes in computer science 3735: 70–83.

[55] Jing Y, Pavlovic V, Rehg JM (2005) Efficient discriminative learning of Bayesian network classifier via boosted augmented naive Bayes. Proc Int Conf Mach Learn 22: 369–376.

[56] Langseth H, Nielsen TD (2006) Classification using hierarchical naïve Bayes models. Mach Learn 63 (2): 135–159.

[57] Su J, Zhang H (2006) Full Bayesian network classifiers. Proc Int Conf Mach Learn 23: 897–904.

[58] O’Donnell RT, Allison L, Korb KB (2006) Learning hybrid Bayesian networks by MML. Advances in artificial intelligence, Lecture notes in computer science 4304: 192–203.

[59] Yehezkel R, Lerner B (2006) Bayesian network structure learning by recursive autonomy identification. Structural, syntactic, and statistical pattern recognition, Lecture notes in computer science 4109: 154–162.

[60] Carvalho AM, Oliveira AL, Sagot MF (2007) Efficient learning of Bayesian network classifiers: an extension to the TAN classifier. Advances in artificial intelligence, Lecture notes in computer science 4830: 16–25.

[61] Boullé M (2007) Compression-based averaging of selective naive Bayes classifiers. J Mach Learn Res 8: 1659–1685.

[62] Francois OCH (2008) Efficient Bayesian network learning using EM or pairwise deletion. European Workshop on Probabilistic Graphical Models 4: 121–128.

[63] Jing Y, Pavlovic V, Rehg JM (2008) Boosted Bayesian network classifiers. Mach Learn 73 (2): 155–184.

[64] Madden MG (2009) On the classification performance of TAN and general Bayesian networks. Knowledge-Based Systems 22 (7): 489–495.

[65] Silander T, Roos T, Myllymaki P (2010) Learning locally minimax optimal Bayesian networks. Int J Approx Reason 51 (5): 544–557.

[66] Drugan MM, Wiering MA (2010) Feature selection for Bayesian network classifiers using the MDL-FS score. Int J Approx Reason 51 (6): 695–717.

[67] Lee N, Kim JM (2010) Conversion of categorical variables into numerical variables via Bayesian network classifiers for binary classifications. Comput Stat Data Anal 54 (5): 1247–1265.

[68] Flores MJ, Nicholson AE, Brunskill A, Korb KB, Mascaro S (2011) Incorporating expert knowledge when learning Bayesian network structure: a medical case study. Artif Intell Med 53 (3): 181–204. 10.1016/j.artmed.2011.08.004 21958683

[69] Flores MJ, Gámez JA, Martínez AM (2011) Handling numeric attributes when comparing Bayesian network classifiers: does the discretization method matter? Applied Intelligence 34 (3): 372–385.

[70] Larrañaga P, Karshenas H, Bielza C, Santana R (2013) A review on evolutionary algorithms in Bayesian network learning and inference tasks. Information Sciences 233: 109–125.

[71] Friedman N, Goldszmidt M (1998) Learning Bayesian networks with local structure. Learning in graphical models, NATO ASI series 89: 421–459.

[72] Quinlan JR (1986) Induction of decision trees. Mach Learn 1: 81–106.

[73] Neapolitan RE (2004) Learning Bayesian networks. New Jersey: Pearson-Prentice Hall. 674 p.

[74] Isozaki T (2012) Learning causal Bayesian networks using minimum free energy principle. New Generation Computing 30 (1): 17–52.

[75] Drugan MM, Wiering MA (2010) Feature selection for Bayesian network classifiers using the MDL-FS score. Int J Approx Reason 51 (6): 695–717.

[76] Lee N, Kim JM (2010) Conversion of categorical variables into numerical variables via Bayesian network classifiers for binary classifications. Comput Stat Data Anal 54 (5): 1247–1265.

[77] Alonso-Barba JI, delaOssa L, Puerta JM (2011) Structural learning of Bayesian networks using local algorithms based on the space of orderings. Soft Computing 15 (10): 1881–1895.

[78] Freno A, Trentin E (2011) Hybrid random fields: a scalable approach to structure and parameter learning in probabilistic graphical models. Berlin, Germany: Springer Berlin-Heidelberg. 207 p.

[79] Lima CF, Lobo FG, Pelikan M, Goldberg DE (2011) Model accuracy in the Bayesian optimization algorithm. Soft Computing 15 (7): 1351–1371.

[80] Liu Y, Han JDJ (2010) Application of Bayesian networks on large-scale biological data. Front Biol (Beijing) 5 (2): 98–104.

[81] de Campos CP, Zeng Z, Ji Q (2009) Structure learning of Bayesian networks using constraints. Proc Int Conf Mach Learn 26: 113–120.

[82] Yang Y, Webb G, Korb K, Ting KM (2007) Classifying under computational resource constraints: anytime classification using probabilistic estimators. Mach Learn 69 (1): 35–53.

[83] Sahin F, Tillett J, Raghuveer R, Rao T (2004) An evolutionary algorithmic approach to learning a Bayesian network from complete data. Proc. SPIE 5433, Data Mining and Knowledge Discovery: Theory, Tools, and Technology VI: 88–99.

[84] de Campos LM (2006) A scoring function for learning Bayesian networks based on mutual information and conditional independence test*s.* . J Mach Learn Res 7: 2149–2187.

[85] Cheng J, Greiner R, Kelly J, Bell D, Liu W (2002) Learning Bayesian networks from data: an information theory based approach. Artif Intell 137: 43–90.

[86] Ide JS, Cozman FG (2002) Random generation of Bayesian networks. Advances in artificial intelligence, Lecture notes in computer science 2507: 366–376.

[87] Press WH, Flannery BP, Teukolsky SA, Vetterling WT (1999) Numerical recipes in C: the art of scientific computing. New York: Cambridge University Press. 994 p.

[88] Hastie T, Tibshirani R, Friedman J (2001) The elements of statistical learning. New York: Springer. 533 p.

[89] Friedman N, Yakhini Z (1996) On the sample complexity of learning Bayesian networks. Uncertain Artif Intell 12: 274–282.

